# A New Chronology for the Bronze Age of Northeastern Thailand and Its Implications for Southeast Asian Prehistory

**DOI:** 10.1371/journal.pone.0137542

**Published:** 2015-09-18

**Authors:** Charles F. W. Higham, Katerina Douka, Thomas F. G. Higham

**Affiliations:** 1 Department of Anthropology and Archaeology, University of Otago, Dunedin, New Zealand; 2 Oxford Radiocarbon Accelerator Unit, Research Laboratory for Archaeology and the History of Art, University of Oxford, Oxford, United Kingdom; New York State Museum, UNITED STATES

## Abstract

There are two models for the origins and timing of the Bronze Age in Southeast Asia. The first centres on the sites of Ban Chiang and Non Nok Tha in Northeast Thailand. It places the first evidence for bronze technology in about 2000 B.C., and identifies the origin by means of direct contact with specialists of the Seima Turbino metallurgical tradition of Central Eurasia. The second is based on the site of Ban Non Wat, 280 km southwest of Ban Chiang, where extensive radiocarbon dating places the transition into the Bronze Age in the 11th century B.C. with likely origins in a southward expansion of technological expertise rooted in the early states of the Yellow and Yangtze valleys, China. We have redated Ban Chiang and Non Nok Tha, as well as the sites of Ban Na Di and Ban Lum Khao, and here present 105 radiocarbon determinations that strongly support the latter model. The statistical analysis of the results using a Bayesian approach allows us to examine the data at a regional level, elucidate the timing of arrival of copper base technology in Southeast Asia and consider its social impact.

## Introduction

Two conflicting chronological models exist for the Bronze Age of Southeast Asia [1,2]. The presence of an independent Bronze Age in Southeast Asia was first identified and evaluated in the 1870s in the wake of the establishment of a French protectorate over the Kingdom of Cambodia. Centred on the settlement of Samrong Sen, the tin-bronze axes, bangles and fish hooks were dated in the later first millennium BC [3,4]. Further prehistoric bronzes were recovered with the expansion of fieldwork into Laos [5]. In the 1960s, a stratigraphic sequence spanning the Neolithic into the early Bronze Age was identified at the northeastern Thai site of Non Nok Tha [6], confirmed in 1974–5 at Ban Chiang [7].

Attempts to build a chronological framework for the Southeast Asian Bronze Age and thereby understand the social impact of copper base technology in the region resulted in controversy. Solheim [8] and Gorman and Charoenwongsa [7] claimed a fourth millennium B.C. date for the sites of Non Nok Tha (102° 18´ 17˝ E, 16° 47´ 57˝ N) and Ban Chiang (103° 14´ 23˝ E, 17° 24´ 23˝ N). Where some scholars accepted a chronological context so early as to require an indigenous origin for copper-base metallurgy, others remained highly sceptical [9]. Furthermore, the lack of any significant evidence for social change towards a controlling and elite group with the advent of metallurgy at either Non Nok Tha or Ban Chiang has confounded expectations [10].

More recently, other authors [1] have placed the transition from the Neolithic into the Bronze Age at the site of Ban Chiang between 2000–1800 B.C. (the long chronology model, abbreviated LCM), while Higham and Higham have applied a Bayesian analysis to a suite of 76 radiocarbon determinations from Ban Non Wat [2], which supported a much later 11th century B.C. transition into the Bronze Age (short chronology model, abbreviated SCM).

This gap of nearly 1000 years, or ~50 human generations, between the two proposed hypotheses is of more than regional interest. Resolution of the issue has profound implications on theories of the cultural transfer of knowledge and the impact of metallurgy on society. The LCM has led to the proposal that the knowledge of mining, smelting and alloying copper and tin was transmitted in a complete manner by experienced practitioners trained in the Seima-Turbino technological system of Central Asia and Siberia, a phenomenon expedited inter alia by societies in which there was modest social differentiation and no elite jealous of securing privileged access to valuables, including those cast in bronze [1]. This model of long-distance movement of metal working knowledge is based on dating evidence obtained from the site of Ban Chiang. White and Hamilton [1] claim support for the LCM by citing Bayard’s [6] placement of the transition to the Bronze Age at Non Nok Tha “*some time after 2500 B*.*C*.”. They also highlight the presence of a bronze bar at Ban Mai Chaimonkol in Central Thailand, a site vaguely dated on the basis of ceramic typology parallels with other sites for which there is as yet no assured chronology. Finally the presence of bronze waste and traces of bronze in some Phung Nguyen culture sites in northern Vietnam has been seen as supportive of the LCM despite Nishimura’s suggestion that “*the absence of casting moulds and complete bronze artefacts in Phung Nguyen sites indicates that the Phung Nguyen is actually the final phase of the Neolithic*” [11]

The second model (SCM) is based on the dated transition into the Bronze Age at Ban Non Wat in the late 11th century B.C. This chronological context harmonises with the long-established and relatively gradual spread of metallurgical skills southward from the Central Plains states of the Yellow River, and is supported by the parallels in casting technology between donor and recipient groups [12,13]. Moreover, the early Bronze Age cemetery at Ban Non Wat contained the burial ground of an elite segment of society with, it has been argued, privileged access to exotic items, including axes and ornaments cast in copper [14].

## Previous Chronological Attempts

Chronology is at the heart of the issue. The first attempts at dating Ban Chiang and Non Nok Tha involved the recovery and accumulation of fragments of charcoal from grave fill and occupation contexts until there was sufficient material to warrant a conventional radiocarbon determination, which at the time required several grams of charred plant material. It is not surprising that the results were inconsistent. Unspeciated charcoal has unknown inbuilt age, charcoal from the filling of a burial is almost by definition from a disturbed context, and accumulating separate pieces of charcoal into a sample, even if from a discrete hearth, is precarious [15]. In a second attempt at dating these two sites, White [16] and Bayard [6] turned to the AMS dating of the organic temper in burial and occupation pots. While the vessels are probably in secure cultural context, this technique is seriously vitiated by the presence of an unknown fraction of old carbon in the clay used to fashion the pottery vessel in question [17,18]. Six determinations employing this technique and one from rice phytoliths, form the foundation for the LCM, according to which bronze casting at Ban Chiang began as early as 2000 B.C. [1].

In 2011, AMS radiocarbon determinations from Ban Non Wat were obtained from well contexted charcoal, and from freshwater bivalve shells placed as mortuary offerings with the dead [2]. It would have been preferable to extract and date collagen from the prehistoric human bones, but none has survived. This is not uncommon in Southeast Asia where soil conditions, as well as high temperature fluctuations and abundance of rainfall and groundwater percolation, create unfavourable conditions for the preservation of organic biomolecules, such as bone collagen. Freshwater shell may sometimes exhibit reservoir effects where the bedrock is of carbonaceous origin and feeds rivers and lakes. In this instance, however, there was no evidence of reservoir offsets, either in the modern analysed freshwater shell or in the archaeological samples where charcoal-shell comparisons from the same contexts were performed.

The Bayesian analysis of the complete set of determinations from Ban Non Wat showed that the charcoal and shell results were in good agreement without any major outliers [2] (S1 Fig). They revealed that the initial Neolithic settlement of the site took place in the 17th century B.C., with the transition into the Bronze Age in the late 11th century B.C., followed immediately by a phenomenal rise in the wealth of those interred that is unparalleled in Southeast Asia [19].

Faced with this gulf of a millennium between the dates for early bronze from two sites (Ban Chiang and Ban Non Wat) located in the same region, we initially dated 5 pig bones and one bovid bone that had been placed with the dead at Ban Chiang (Table A in S1 File). One of us (CFWH) was a member of the excavation team there in 1974–5, and the faunal remains under his analysis were archived and readily available [20]. These first results strongly supported the SCM. A further 10 determinations were later obtained from human bones that span the late Neolithic and early Bronze Age at Ban Chiang; these also dated the earliest grave with a bronze artefact in the 10th century BC [21]. Since 2013 we have undertaken a major research programme to establish the fundamental chronological framework for the later prehistory of the Khorat Plateau in Northeast Thailand, using the most recent dating procedures and interpretative protocols. We report on the new results below.

## Material and Methods

We radiocarbon dated human bone from three of the sites described below at the Oxford Radiocarbon Accelerator Unit (ORAU). Prior to extensive sampling of human skeletal remains we screened small (3–5 mg) sub-samples of drilled bone powder by measuring the elemental nitrogen concentration. This is a useful proxy for protein, and therefore presence of collagen in the bone. We report these results in the Supplementary Methods (Table M in S1 File). Samples with >~0.5–0.7%N were passed for full collagen extraction treatment for AMS dating. The methods used are outlined in Brock et al. [22]. Initially collagen was extracted using an acid-base-acid procedure followed by gelatinization and lyophilisation [22]. Where sufficient gelatin remains, the Oxford preparative method includes a final ultrafiltration pretreatment using a pre-cleaned Vivaspin™30kD MWCO ultra-filter [23,24]. This removes low molecular weight contaminants and produces a better purified collagen fraction as indicated by improved C:N atomic ratios and carbon mass on combustion. The filtered collagen was freeze-dried and combusted in a CHN analyser linked in continuous flow mode to a Europa isotope ratio mass spectrometer (EA-CF-IRMS) using a He carrier gas. We measured δ^15^N and δ^13^C values (reported in ‰), nitrogen and carbon content and also calculated the bone C:N atomic ratios. The purified CO_2_ was then reduced to graphite using H_2_ in a reaction catalyzed by 2 mg of a Fe powder at 560°C for 6 hr. The graphite was pressed into an Al target holder prior to radiocarbon measurement using AMS [25].

Where bone collagen was >1% wt. collagen we gave OxA-X- prefixes as opposed to OxA- prefixes to denote them (with the exception of 3 cases from Ban Chiang). We tested the reliability of dating bone with collagen yields of this size and the models showed that almost none were outliers. All other analytical parameters measured, including the carbon to nitrogen atomic ratio, were acceptable. We therefore consider the results to be robust.

No collagen has survived in the human bones from the site of Ban Lum Khao, so we turned to in-situ mortuary offerings with a limited likelihood of inbuilt age. These involved shell beads and freshwater bivalve shells worn or placed with the dead.

Shell remains were measured both at Oxford and the Waikato Radiocarbon Laboratory. We dated both shell ornaments, in the form of minute (ø 3–4mm) disc-shaped beads, as well as much larger freshwater bivalve shells. The protocols used to extract and measure radiocarbon from shell samples are similar at both laboratories and described below. Initially shell surface contamination was removed using an air abrasion system at Oxford, or washing in diluted HCl at Waikato. A shell fragment was then tested for recrystallization using the Feigl staining method; when no recrystallization was observed the fragment was crushed and prepared for dating. Approximately 25 mg of sample powder was reacted with 5 mL of 80% phosphoric acid (H_3_PO_4_) for 12 hr at 60°C, under vacuum. The CO_2_ evolved via this process was cryogenically purified and transferred into a sealed glass ampoule. The ampoule was cracked, and the gas transferred through the EA-CF-IRMS system, then graphitized and AMS dated using the method described earlier.

In addition, we dated charcoal from the Neolithic occupation of the site of Ban Lum Khao. The ABA protocol used to decontaminate charcoal is well established [22]. In short, an initial HCl step to remove carbonate contamination is followed by a base wash (NaOH) and a final HCl wash. All steps are interspersed by a series of water rinses. The remaining material is freeze-dried, combusted, graphitized and measured as per the methods described previously.

Where samples come from successive phases within a defined cultural sequence, the determinations can be analysed using a Bayesian statistical approach to obtain posterior probability distributions derived from prior archaeological knowledge and the calibrated age ranges. This is crucial information in the interpretation of the chronology of a particular site and its formation, since it provides statistically-calculated age ranges for otherwise undated events, for example the boundary transitions between archaeological phases identified in the field, and the duration of these phases. Examples in the archaeological literature are now standard [26,27].

**Fig 1:**
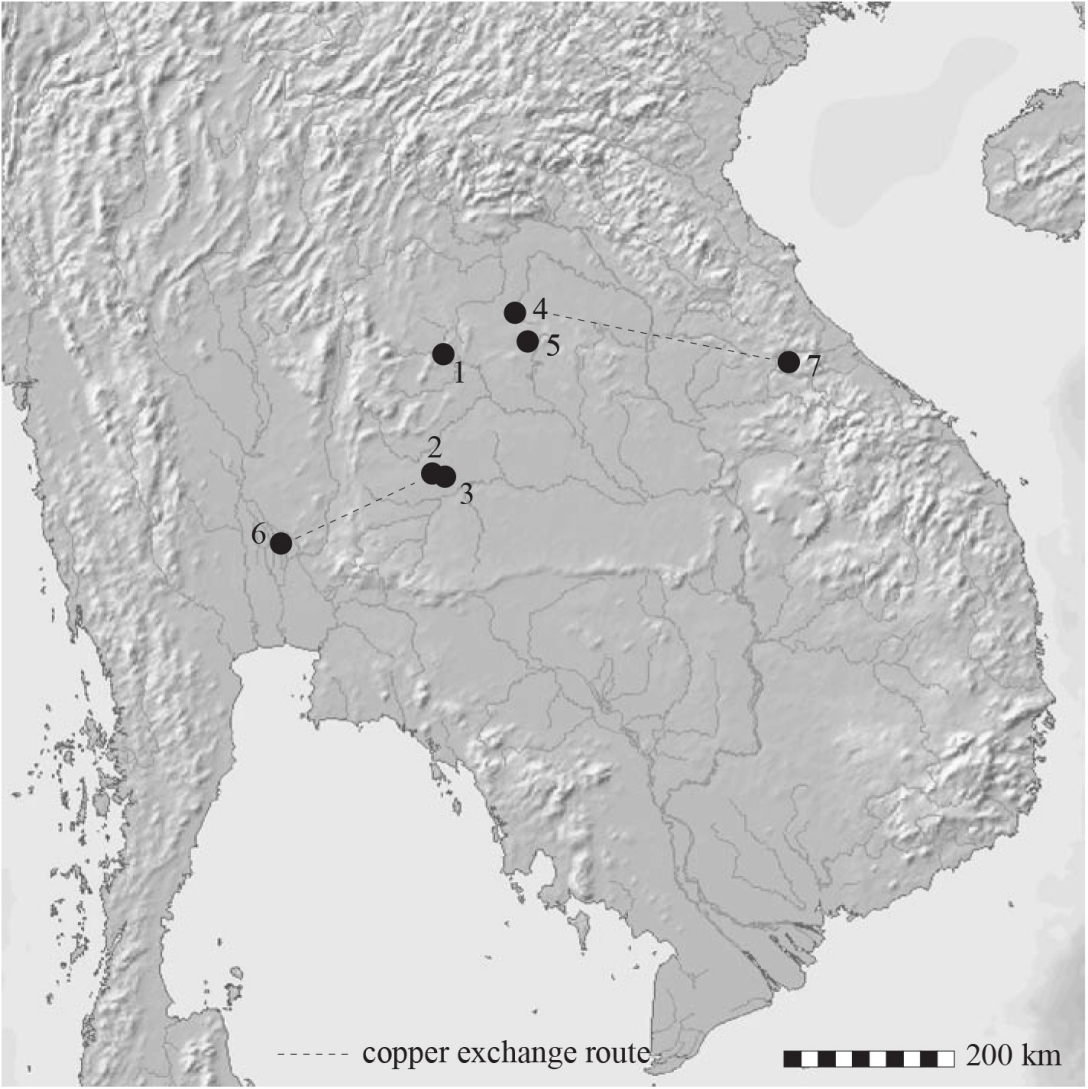
Topographic map with the location of the Thai sites mentioned in the text. 1. Non Nok Tha, 2. Ban Non Wat, 3. Ban Lum Khao, 4. Ban Chiang, 5. Ban Na Di, 6. Khao Wong Prachan, 7. Xepon. Dashed line indicates copper exchange networks between sites and mines. Scale 200 km.

Satisfactory resolution of the dating impasse regarding the start of the Bronze Age in Southeast Asia requires sites in which there was a transition between the Neolithic and Bronze Ages. The cultural phases in the Thai sites in this study are, in the main, defined on the basis of the superposition of burial groups. Further relevant information can then come from other sites in which there is a suite of determinations either from Neolithic or the Bronze Age contexts. In addition to the fully published radiocarbon chronology for Ban Non Wat [2], we have identified and analysed four further sites on the Khorat Plateau of Northeast Thailand: Ban Chiang, Non Nok Tha, Ban Lum Khao (102° 20´ 35˝ E, 15° 14´ 56˝ N) and Ban Na Di (103° 8´ 2˝ E, 17° 15´ 22˝ N) (Fig 1).

### Ban Chiang

Two areas of Ban Chiang were excavated in 1974–5. Both incorporated superimposed Neolithic, Bronze and Iron Age burials. Ten phases have been identified [28,29]. Early Periods (EP) I-II are Neolithic. In the 1974 excavation square, some fragments of bronze were found in EP II layers. It has been argued that these indicate the presence of bronze metallurgy at that juncture [1]. Best practice, however, restricts acceptance of evidence for a bronze industry to an in situ casting furnace, or a clear mortuary association, such as bangles on a wrist.

**Fig 2:**
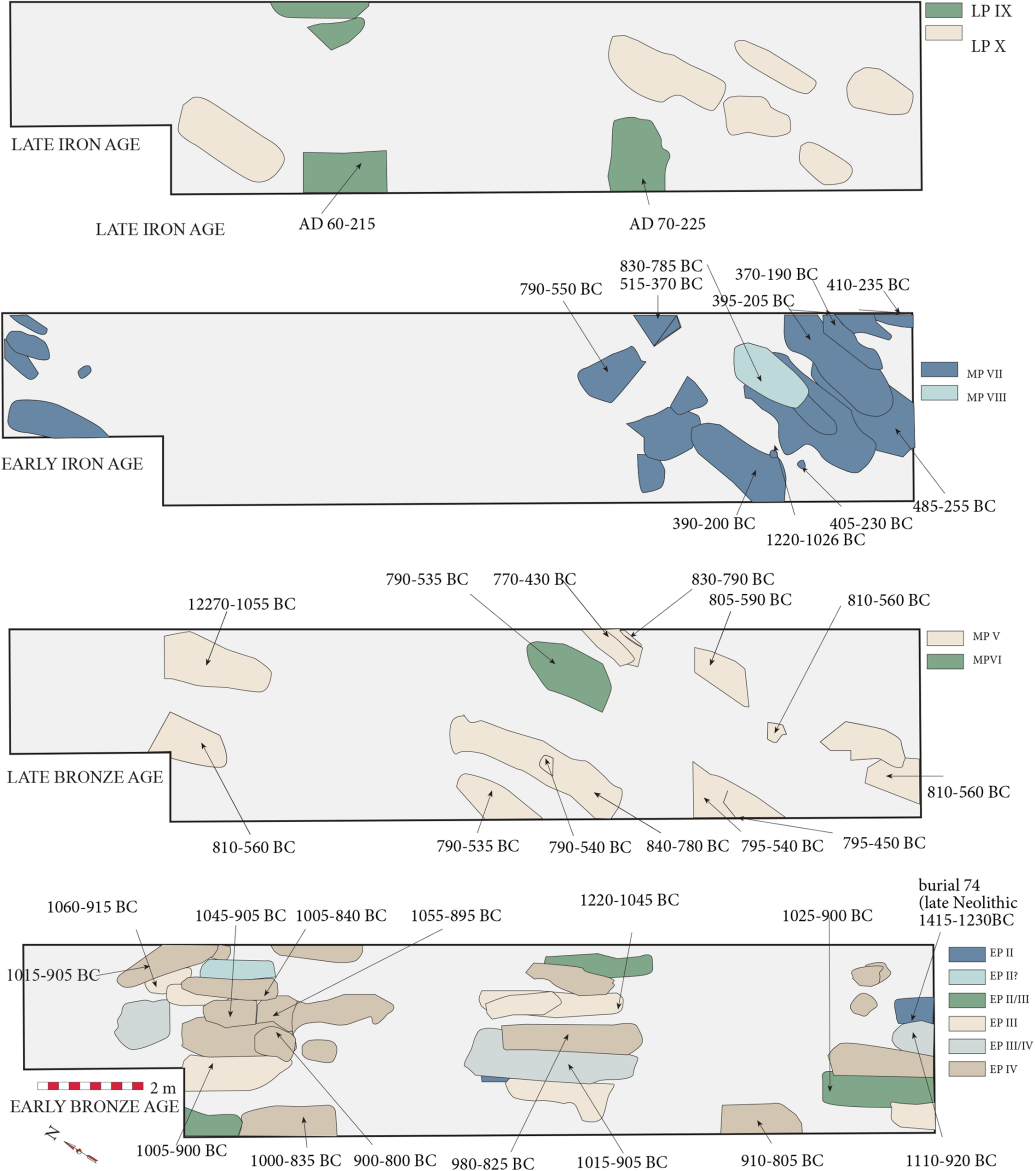
The layout of the burials from the 1975 excavation at Ban Chiang, together with the new calibrated AMS radiocarbon determinations, at 95.4% probability. The date for burial 74 (1415–1230 BC) is omitted because it is assigned to the late Neolithic EP II.

Burial 72 of EP II/III had a flat piece of bronze at the base of the grave. EP III provided the earliest burial with a finished bronze artefact in the form of a socketed spear with burial 76. EP III-IV represent the earlier Bronze Age, which, in the 1975 square, comprised three separate rows of burials (Fig 2). A later set of burials on a different orientation has been assigned from EP V to Middle Period (MP) VIII. EP V-MP VI represent the later Bronze Age, while MP VII saw the first presence of iron artefacts in a burial. The Late Period (LP) belongs to the later Iron Age. In the absence of a site report for Ban Chiang, we have no plan for the 1974 season burial, except that there was just one EP I burial, and several assigned to EP II.

We have taken samples of bone from 51 burials excavated at Ban Chiang, of which 44 yielded sufficient collagen for AMS dating. Some burial contexts were dated twice. Four pig bones associated as mortuary offerings were also dated. These came from articulated bones in direct association with the human remains.

### Non Nok Tha

Non Nok Tha is the second site at the heart of the LCM [1]. The cultural sequence at this cemetery site has been divided by Bayard into 11 phases [6]. The first two (EP 1–2) are Neolithic. Just one late EP 3 grave contained a bronze offering comprising a typologically very early form of socketed axe. MP 1–8 comprised burials assigned to the Bronze Age on the basis of associated bronzes, crucibles and moulds with the dead. We sampled all the available skeletons from the 1966 and 1968 seasons, and the 15 that provided sufficient collagen for AMS dating cover the sequence from the initial Neolithic settlement to the end of the Bronze Age (S2 Fig).

### Ban Lum Khao

Ban Lum Khao [30] has a four-phase sequence beginning with a Neolithic occupation, then a set of late Neolithic burials, which was followed by an early and a late Bronze Age cemetery (S3 Fig). For the Neolithic occupation, we obtained radiocarbon determinations from pits containing large quantities of charcoal [31]. We recognize that the results might be affected by inbuilt age so each is regarded as a *terminus post quem* in the Bayesian analysis and is also assigned a “charcoal outlier model” which allows for an inbuilt age to be taken into consideration. We attempted unsuccessfully to extract collagen from the human bones. Instead we have dated the early Bronze Age cemetery on the basis of the bivalve shells and disc-shaped beads that were placed as mortuary offerings. The latter, if produced from marine shell, would exhibit reservoir effect and require appropriate correction during calibration. However, such an offset (~400 yr) was not observed amongst the results obtained from charcoal and shells, which are indistinguishable. It was not always possible to be certain of a primary and contemporary association between a bivalve shell with a burial if the latter had been disturbed.

### Ban Na Di

Ban Na Di is a later Bronze and Iron Age site located just 19 km southwest of Ban Chiang. It was excavated in two areas [32]. Both contained Bronze Age graves, some of which were superimposed, and which have been divided into mortuary phases 1a, 1b and 1c. In some cases, for example with burial 15 where there is no superposition with any other burial, the precise place in the sequence is not readily identified. Hence, integrating the burials from the two areas is not straightforward. On the basis of parallels in ceramic forms and decoration, this sequence corresponds to the later Bronze Age at nearby Ban Chiang. Previous radiocarbon determinations at Ban Na Di came from securely provenanced charcoal samples, some deriving from bronze casting furnaces. These determinations originally suggested that the proposed dates at Ban Chiang were erroneously early [33]. Here, we obtained collagen from eight skeletons at this site and produced nine new AMS determinations (burial 28 was dated twice).

A disclaimer with the catalogue numbers of all analysed material and its current location can be found after the Acknowledgments section.

## Results

### Ban Chiang

**Fig 3:**
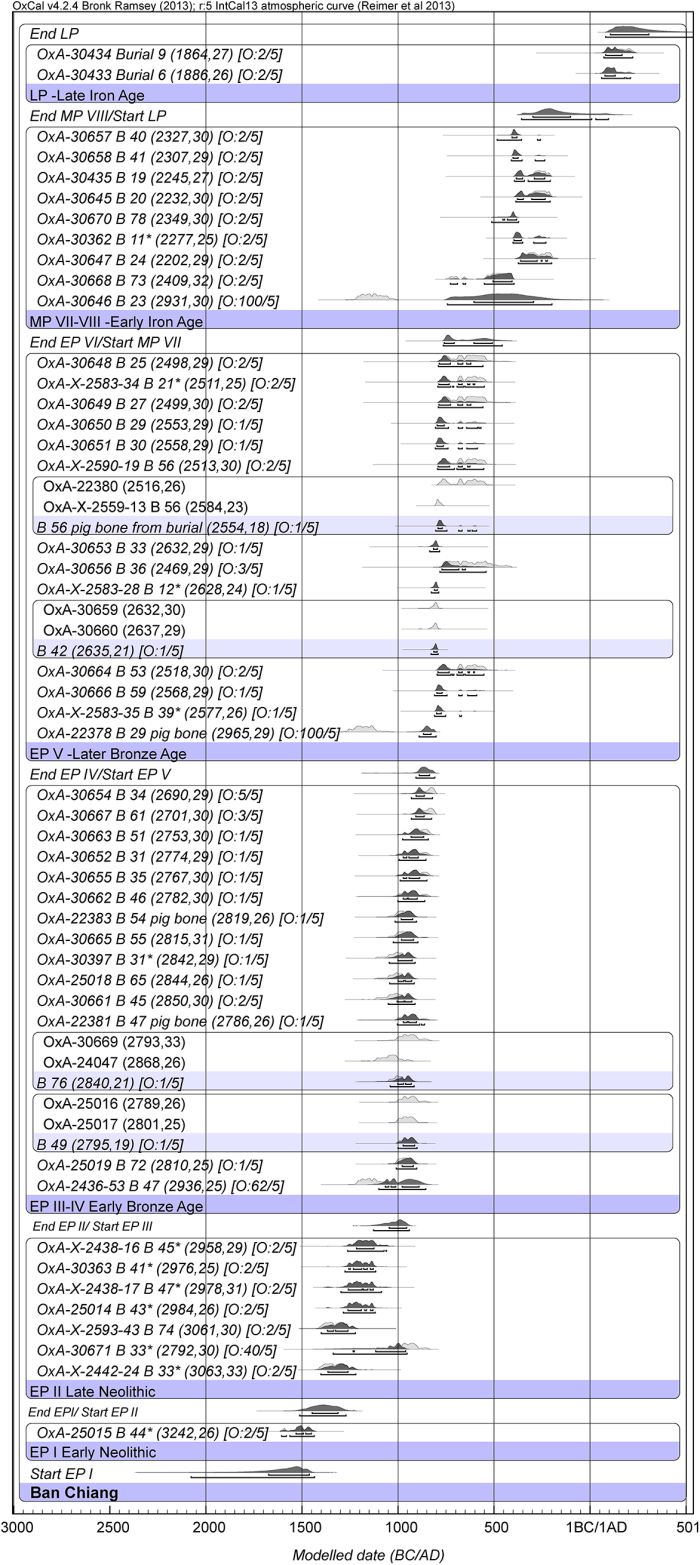
Bayesian age model for the site of Ban Chiang. (OxCal. v4.2.4 Bronk Ramsey (2009 [38]); r:5 IntCal13 atmospheric curve (Reimer et al. 2013 [39]).

We obtained 54 radiocarbon determinations from the Neolithic, Bronze Age and Iron Age phases of the sequence (Tables A, E, I in S1 File). In the Bayesian analysis of the radiocarbon determinations from human bones, in the main, and four pig bones (Fig 3), the burials from EP I-II from the 1974 excavated area have been combined with all the dated burials from the 1975 trench.

The results demonstrate the following dated sequence for this site. There is only one EP I burial, and it has been dated between 1600–1450 BC (at 68.2% probability). The succeeding EPII Neolithic span has been placed in the 13th to the 11th centuries BC. Burial 72 from EP II-III has been cited as evidence for the LCM by the presence of a flat piece of bronze at the bottom of the grave [1]. The date of bone collagen for this burial is 1000–925 BC (at 68.2%). The transition from the late Neolithic to Bronze Age EP III took place between 1050–955 BC. Burial 76, which contains the earliest bronze artifact at the site, has provided two statistically identical determinations ranging between 1110–1000 and 995–905 BC (the statistically combined age is 1025–935 BC). The early Bronze Age cemetery has been divided into EP III to IV. Its duration was brief according to the spread of radiocarbon dates, ranging between 80–220 years (at 68.2% prob.) or between 3–7 human generations, and is placed in the main in the 10th century BC. The later Bronze Age EP V starts at some point between 890–835 BC and covers mainly the 8^th^ to the 7^th^ centuries BC (interval 60–375 years (68.2% prob.)). The MP VII Iron Age starts between 760–505 BC and covers the 3^rd^ and 4^th^ centuries BC. The determination for burial 23 (1210–1055 BC) is clearly too early for this period. This burial comprised disturbed upper limbs with bronze bangles ([28]:425). It is suggested that these bones might have been misplaced in the sequence or re-deposited from a lower level and are out of their original context. Two burials from the late Iron Age are dated to the first two centuries AD.

These new results have made necessary a detailed consideration of the dating methodology used to produce the previous determinations on organic clay on which the LCM is based [16,29]. There are two series of determinations, the first from the Oxford laboratory, the second from Arizona. The radiocarbon data for the former were obtained using two principal pretreatments. The first involved teasing fragments of rice chaff out of the pottery matrix and then treating the sample with acetone, 0.2 N HCl and 0.5 N NaOH successively to extract lipids, carbonates and humic acids. In the second pretreatment the above procedure was applied to crushed potsherds, and followed by either a mixture of 4 M HF in 6 M HCl or concentrated HF followed by multiple washings. This procedure “*aims to concentrate the carbon content of the residue and also makes soluble a considerable quantity of clay-bound humic material*” [29].

In their comments on these determinations, Glusker and White had to confront radically different results for the same pottery vessel. Thus for burial 47 of EP III, the HF pretreatment on crushed potsherd residues resulted in a determination of 4810±90 BP, while the date from rice fragments was 2910±90 BP [29]. Glusker and White commenting on all four cases of results obtained from rice chaff rather than crushed sherds, found them “*younger than archaeological expectations*” ([29]:260), and have subsequently set them aside from further consideration. Yet all four fit perfectly into our new sequence derived from human bone collagen.

The second series of AMS determinations from clay organics comes from the University of Arizona laboratory. The sherds were first crushed to 2–3 mm particle size, then “*subjected to successive extractions with acetone (to extract lipids)*, *0*.*2N HCl (to extract carbonates) and 0*.*5N NaOH (to extract humic acids)*, *with multiple washings in between*. *The residue was further subjected to a mixture of 4N HF in 6M HCl or concentrated HF (49%) followed by multiple washings*” ([34]:104). One further determination on rice phytoliths was obtained from the Lawrence Livermore National Laboratory (CAMS-41264).

The proponents of the LCM selected for establishing the chronology of Ban Chiang, the sample of rice phytoliths and six determinations from the second pretreatment undertaken at Arizona. In the Oxford series, these invariably yields the earliest results when both were applied to the same pot. All 12 Oxford AMS results were excluded from consideration ([16]:97). However, when compared with the direct dates on bone collagen from the human burials, these rather experimental determinations on clay are clearly erroneous. We suggest that the offsets are due to; 1) problems with the preparation of the pottery samples prior to dating, specifically a high combustion temperature which does not allow for the removal of a labile volatile fraction of the clay which often has a different radiocarbon age, and; 2) the fact that the clay used to fashion pots may, by its very nature, contain an unquantified amount of old carbon of unknown origin; 3) similarly, the clay-bound humic material may have several sources of origin and C content [17,18].

**Fig 4:**
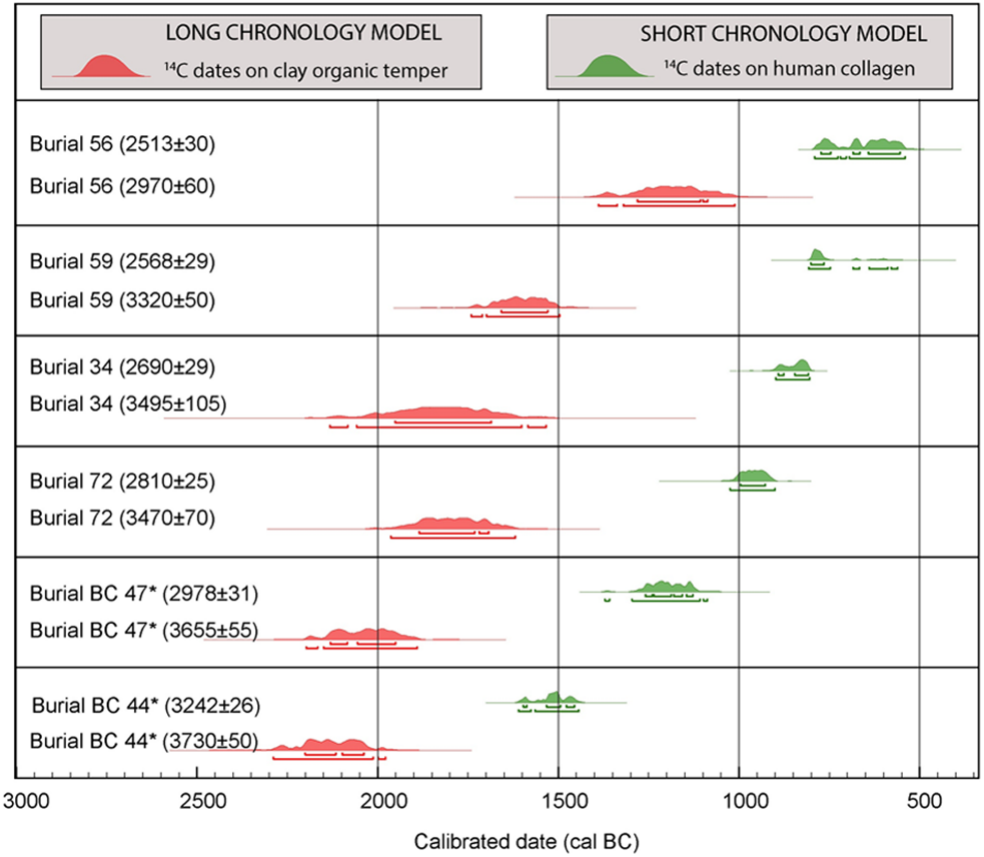
The offsets between previous and current radiocarbon determinations for the same Ban Chiang burials on the basis of clay temper (in red) and human bone (in green).

We find that the new set of AMS radiocarbon determinations on bone collagen show significant differences when compared with the associated ceramic vessels from the same grave that form the basis of the LCM (Fig 4). Burial 44 is the earliest burial encountered. The radiocarbon determination from rice phytoliths is up to 600 years earlier than the AMS date of collagen from the actual burial. In the case of EP II burial 47, the offset between the bone collagen and the clay temper determination is approximately 1000 years, while burial 59 in EP V has a collagen date a millennium later than the temper determination.

### Non Nok Tha

**Fig 5:**
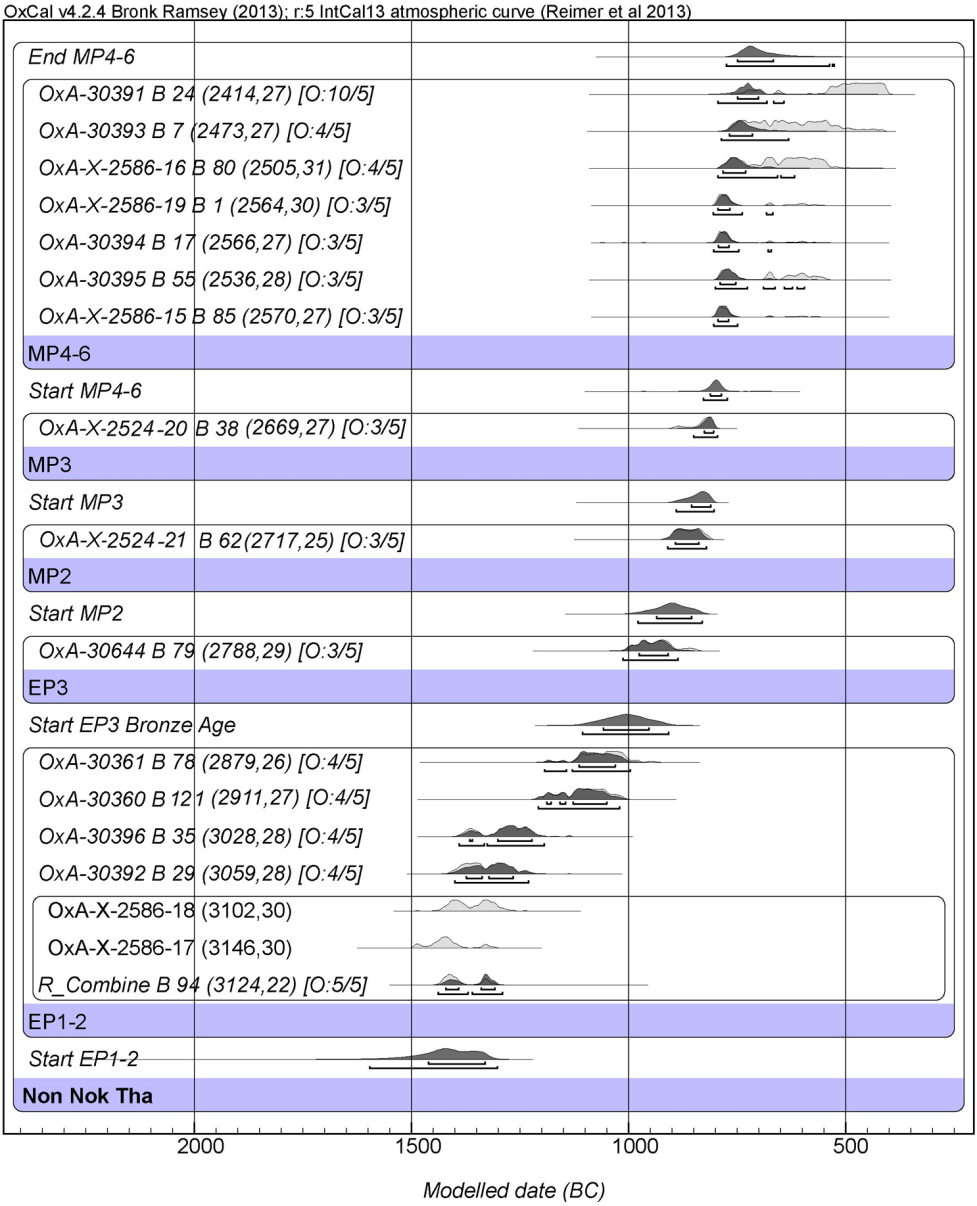
Bayesian age model for the site of Non Nok Tha. (OxCal. v4.2.4 Bronk Ramsey (2009 [38]); r:5 IntCal13 atmospheric curve (Reimer et al. 2013 [39]).

This site’s previous radiocarbon chronology has also been used to support the LCM for the start of the Bronze Age [1]. We have dated 15 burials and obtained 16 AMS dates (one was dated twice) covering the Neolithic and Bronze Age cemeteries, again using human bone collagen (Fig 5, Tables B, F, J in S1 File) [35]. Burial 94, which represents EP 1, the earliest Neolithic mortuary phase, has been dated twice to ~ 1500–1300 BC at 68.2%. EP 2, the second Neolithic mortuary phase, ended between 1050–950 BC. Burial 79 of EP 3, the phase with the earliest socketed copper base axe has been directly dated to the 10^th^ century BC (980–900 BC). For the full Bronze Age, burial 62 from the 1966 season MP 2, has been dated to 900–830 BC. Burial 38 from 1966 MP 3 is dated in the 9^th^ century BC (840–800 BC), while the results from the 1968 season MP 4–6 phase are virtually identical, falling in the 8^th^–6^th^ centuries BC.

### Ban Lum Khao

**Fig 6:**
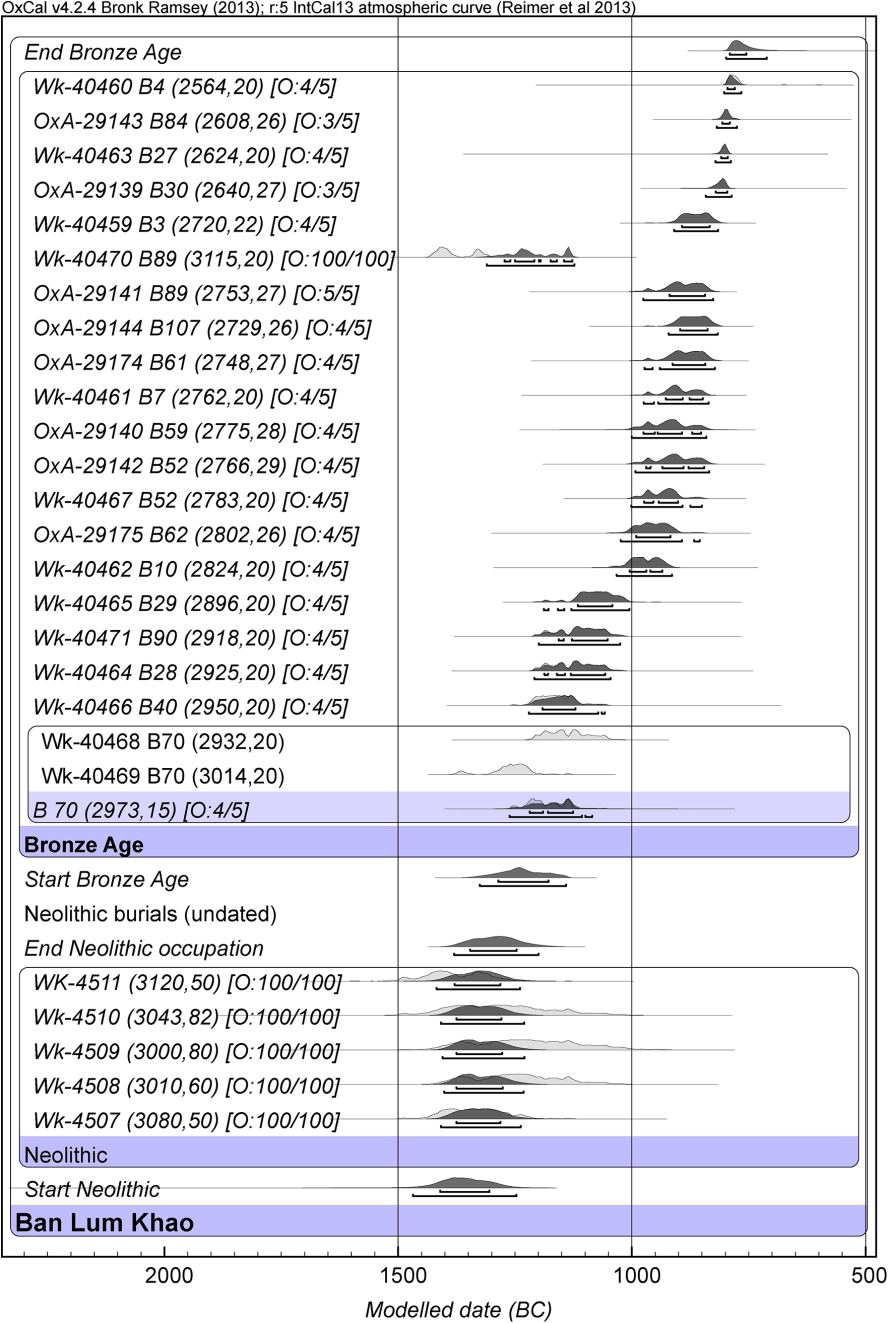
Bayesian age model for the site of Ban Lum Khao. (OxCal. v4.2.4 Bronk Ramsey (2009 [38]); r:5 IntCal13 atmospheric curve (Reimer et al. 2013 [39]).

Our initial dating of this site involved only the basal Neolithic occupation, which is placed between 1400–1300 BC (at 68.2%) or 1470–1245 BC (at 95.4%) [31]. A Neolithic cemetery underlying the Bronze Age burials has not been dated, hence our model contains an undated phase to separate the Neolithic pits from the start of the Bronze Age cemetery. The early Bronze Age cemetery at this site furnished many ceramic vessels as mortuary offerings. A detailed analysis of their form has shown a precise parallel, and presumed contemporaneity, with Bronze Age II at Ban Non Wat. We have obtained 26 AMS determinations from this site (Fig 6, Tables C, G, K in S1 File).

As previously mentioned, we were not able to extract collagen from the human burials. Instead, our chronology is based on AMS determinations of shell beads and unworked bivalve shells found in association with human remains. We note that in some cases there is an offset between two determinations from the same grave. In the case of burial 89, for instance, the date for the shell bead conforms with respective dates for the early Bronze Age at Ban Non Wat, but the bivalve shell is much older and hence rendered an outlier by the model (Fig 6). The upper part of the skeleton of burial 89 was very disturbed, and the shell might not be in primary association, or survived as an heirloom [30]. The same is true for burials 29, 40 and 70. The other bivalve shells were found in close association with the skeletons. We ought therefore to consider this material and determinations on it as *termini post quos* only and not as reliable as direct dates on human bones. The presence of a yet-undetectable reservoir offset affecting these shell dates is highly unlikely given the local hydrogeology. With these considerations in mind, the Bayesian analysis for the earliest Bronze Age burials places the start of the early Bronze Age in the last centuries of the second millennium BC (1290–1170 BC) (Fig 6, Table C in S1 File).

### Ban Na Di

**Fig 7:**
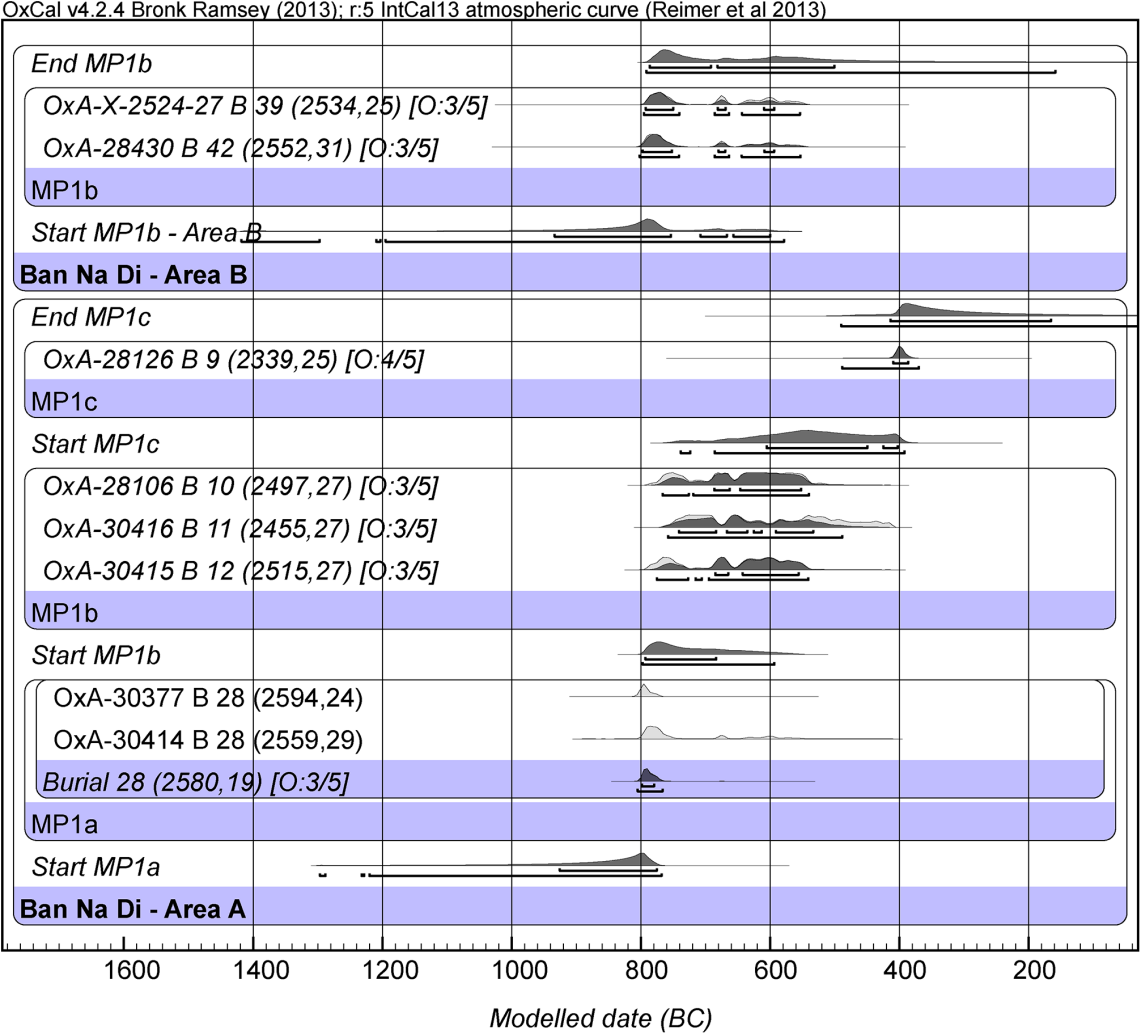
Bayesian age model for the site of Ban Na Di. (OxCal. v4.2.4 Bronk Ramsey (2009 [38]); r:5 IntCal13 atmospheric curve (Reimer et al. 2013 [39]).

As mentioned earlier, Ban Na Di is located in the vicinity of Ban Chiang [32]. The sequence at this site began in the later Bronze Age, and partially overlapped with that documented at Ban Chiang for MP V burials. We have obtained a new series of nine determinations from collagen extracted from human bone. The determinations are virtually identical with those obtained for the equivalent mortuary contexts we have dated on the basis of human bone collagen at Ban Chiang. The two groups of burials come from different parts of the site (Area A and B) and are treated separately in the Bayesian analyses (Fig 7, Tables D, H, L in S1 File).

### The significance of the new results for the Khorat Plateau and wider Southeast Asia

**Fig 8:**
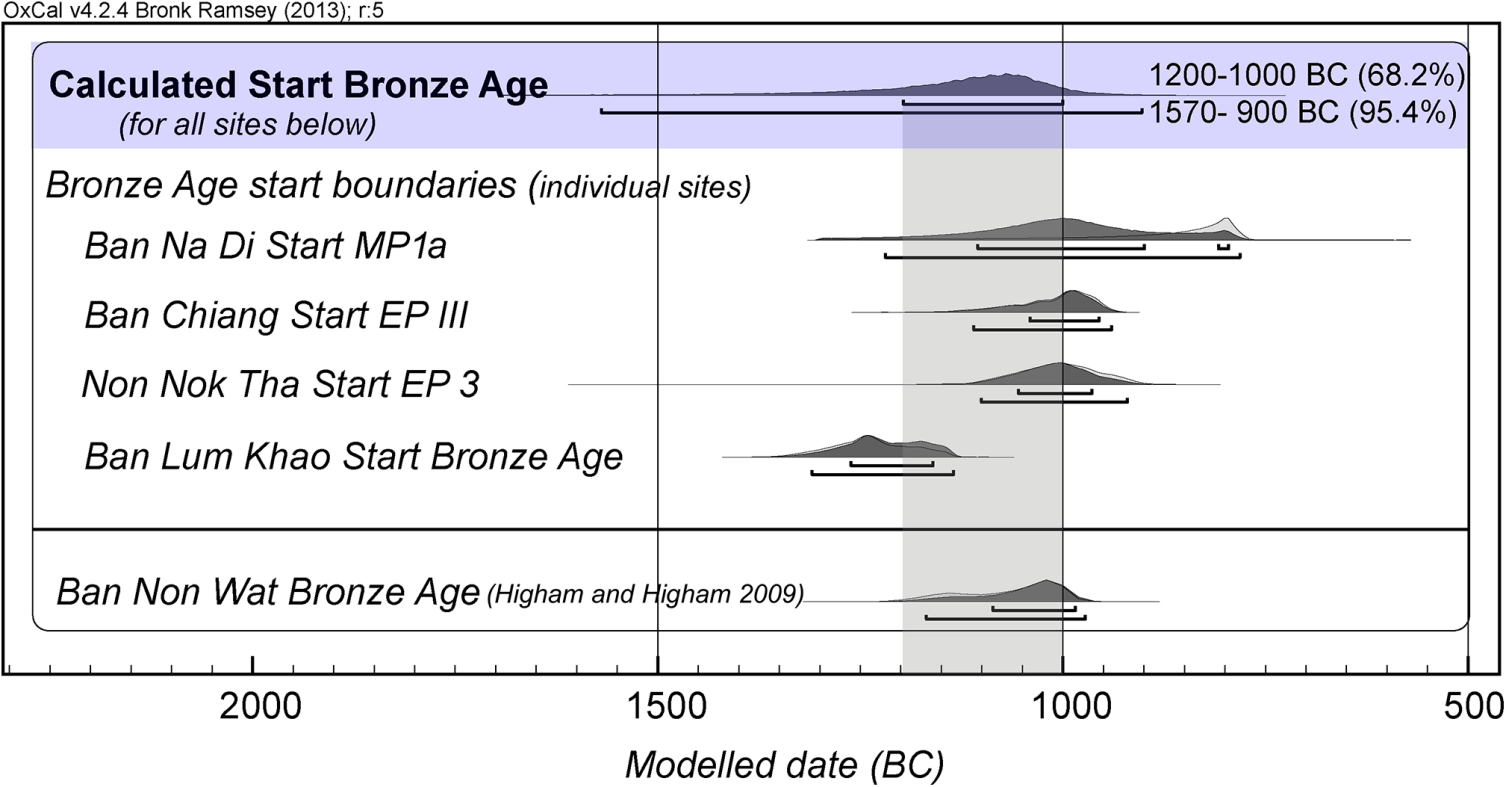
Bayesian probability functions (PDFs) for the beginning of the Bronze Age in Thailand. Using the individual site data for the five dated sites (Ban Chiang, Ban Na Di, Ban Lum Khao, Non Nok Tha, Ban Non Wat) we calculated the probability distribution for the start of the Bronze Age in the region (1200–1000 BC at 68.2%, shaded grey, or 1570–900 BC at 95.4%). Figure was generated using OxCal v4.2.4. and IntCal13.

On the basis of new AMS radiocarbon determinations from human bone collagen, we have demonstrated that Neolithic settlement of the Khorat Plateau began in the investigated sites within the first half of the second millennium BC. Using all available determinations from five well-dated sites (Ban Chiang, Ban Na Di, Ban Lum Khao, Non Nok Tha, Ban Non Wat) we generated an overall likelihood for the transition into the Bronze Age. This took place in the late 11th or the 10th centuries BC (1200–1000 BC at 68.2%, or 1570–900 at 95.4%), lending support to the SCM (Fig 8).

The LCM is based on a handful of erroneous dates for Ban Chiang and Non Nok Tha that place the establishment of the Bronze Age between 2000–1800 BC. This is not supported by the new results on the actual human burials presented above, and must be rejected. Under the LCM, bronze casting in Northeast Thailand was too early for identifying its origins in the dissemination of technical knowledge from the early state societies of China. Therefore, origins were sought further afield, in the Seima-Turbino complex centred between the Dneiper River and the Altai Mountains, the closest site being approximately 4000 km northwest of Ban Chiang [1]. The SCM supports an alternative model [12,13]. There is a reassuring harmony between the timing and the bronze industries of Southeast Asia and Lingnan, southern China, that we have already summarized in detail elsewhere [21]. From Lingnan, the technology of bivalve mould casting can be traced in logical and progressively earlier steps back to the early bronze traditions of the Shang Dynasty.

The establishment of copper base metallurgy on the Khorat Plateau appears to have taken place virtually simultaneously across the several dated sites in the late 11th to the 10th century BC. The refined chronological framework for the onset of the Bronze Age now makes it possible to consider its social impact. Until the excavation of Ban Non Wat, no site provided evidence for the establishment of a social elite. The mortuary evidence rather suggested only slight variations in the ritual treatment of the dead. Under the LCM, the Bronze Age was seen as a lengthy period involving about 100 human generations of social stasis.

The new SCM and the exposure at Ban Non Wat of an early Bronze Age group of outstandingly wealthy burials, including those of infants (S4–S7 Figs), are held to indicate a rapid rise of a hereditary elite that enjoyed preferred access to exotic valuables, such as copper-base ornaments and axes, for several generations [14]. The wealth of the early Bronze Age burials at Ban Non Wat contrasts with the poverty of their contemporaries at Non Nok Tha, Ban Chiang and Ban Lum Khao (S8 Fig). One explanation for this might lie in the strategic location of Ban Non Wat at the eastern portal of a pass that would have brought copper and marine shell from production sites in Central Thailand [36].

The new chronology presented above also provides insights into the exchange routes employed in the early exploitation of copper ores. Copper used to cast the EP III spearhead from Ban Chiang has been sourced to the Xepon mines in upland Laos, indicating mining activity there at least 500 years before the earliest context yet identified at the mining complex itself [37]. Again, copper identified in the earliest Bronze Age at Ban Non Wat has the isotopic signature of the Khao Wong Prachan Valley mines in Central Thailand. Clearly, copper was travelling over considerable distances from different mines. We conclude that copper-base technology spread rapidly across the mainland of Southeast Asia in the late 11th century BC. The coherent chronological framework presented here [38,39], together with the documented casting technology of the founders, dovetails neatly with our knowledge of the initial Bronze Age in Lingnan, southern China.

## Supporting Information

S1 Fig Probability distribution of dates relating to the cultural sequence of Ban Non Wat. (OxCal. v4.2.4 Bronk Ramsey (2009 [38]); r:5 IntCal13 atmospheric curve (Reimer et al. 2013 [39]).
(TIFF)

S2 Fig Plan of the 1968 season cemetery at Non Nok Tha. The dates refer to calibrated, unmodelled ages in years BC at 95.4% probability.
(EPS)

S3 Fig Plan of the early Bronze Age cemetery at Ban Lum Khao. The dates refer to calibrated, unmodelled ages in years BC at 95.4% probability.
(EPS)

S4 Fig Ban Non Wat burial 197, Bronze Age 2. Male individual interred with multiple copper artefacts including socketed axes. The ceramic vessel is decorated with a frieze of dancers. The calibrated AMS determinations for this burial is 1110–915 BC.
(PDF)

S5 Fig Ban Non Wat burial 571, Bronze Age 2. Male individual interred with a copper axe and many ceramic vessels. The dated bivalve shell lies by the head, and the calibrated radiocarbon age is 900–805 BC.
(PDF)

S6 Fig Ban Non Wat burial 263, Bronze Age 3A. Female individual interred with many pottery vessels thought to reflect lavish mortuary feasting. The calibrated radiocarbon age for this burial is 1075–900 BC.
(TIF)

S7 Fig Ban Non Wat burial 290, Bronze Age 2. Male individual interred with three copper socketed axes and a copper chisel. The calibrated radiocarbon age for this burial is 1125–930 BC.
(PDF)

S8 Fig Two dated burials from Ban Lum Khao. The mortuary offerings are much poorer than those at contemporary Ban Non Wat.
(TIF)

S1 File Supplementary Tables. **Radiocarbon determinations from the Ban Chiang site.** Asterisked samples (*) in the context column means material from the 1974 excavation season. The rest of the samples come from the 1975 excavation season. OxA-X- prefixes are given in preference to OxA- numbers when there is a problem with the pre-treatment chemistry, AMS measurement or when there is a novel or experimental protocol applied in the dating. Samples marked with an “S” (^**s**^) are those given a solvent extraction prior to collagen preparation to remove glues or conservatives identified on the bones. Date in this table stands for the conventional radiocarbon age, expressed in years BP. Errors are the determined standard errors (values are ± one standard error). ‘Used’ represents the amount of bone powder pretreated in milligrams. Yield represents the wSeight of collagen or ultrafiltered collagen in milligrams. Yield (%) is the percent yield of extracted collagen as a function of the starting weight of the bone analysed. %C is the carbon present in the combusted collagen. Stable isotope ratios are expressed in ‰ relative to vPDB with a mass spectrometric precision of ±0.2‰ for C and ±0.3‰ for N. C:N is the atomic ratio of C to N and is acceptable if it ranges between 2.9–3.5. ¶ denotes duplicate measurements on the same bone **(Table A). Radiocarbon determinations of human bone from the Non Nok Tha site.** See caption for SI Table for details. ^∫^denotes samples with low collagen yields for which ultrafiltration was not possible (**Table B). Radiocarbon dates from Ban Lum Khao.** Due to the bad preservation of bone collagen from the site, shell and charcoal samples was dated instead of bone. Burial 52 was dated at two labs (Oxford and Waikato) and both determinations exhibit good agreement. Wk-40470 is much older than OxA-29141, both from Bronze Age burial 89; the age of the former shell however is identical to the ages obtained from the Neolithic occupation, hence it is most likely part of the burial infill (disturbed sediment which include material from the lower Neolithic layers) rather than the grave goods. Note that the Neolithic charcoal determinations have much larger standard errors as they were produced using conventional methodologies (**Table C). Radiocarbon AMS dates of human bone from Ban Na Di.** See S1 file table A caption for details. ^∫^ denotes samples treated until the gelatinization step and not ultrafiltration was applied due to low collagen yield. ¶ denotes autoduplicate dates, i.e. repeat dates of the same bone (**Table D). Results of the Bayesian modelling of the Ban Chiang sequence**. Bold titles show the names of the successive phases. Italic scripts denote the calculated boundaries. Radiocarbon likelihoods (simple calibrated ages) are shown in the ‘Unmodelled’ columns, the ‘Modelled’ column shows the posterior probability ranges for each part of the main model. Convergence values are also shown (**Table E). Results of the Bayesian modelling from the site of Non Nok Tha.** See S1 File table A caption for details of the values in this table (**Table F). Results of the calibration and the Bayesian modelling from the site of Ban Lum Khao.** See Caption for S1 File table E for details of the values in this table (**Table G).**. **Results of the Bayesian modelling of the Ban Na Di site, Area A (bottom) and Area B (top).** See caption to S1 file table E for details. Burial 15, excavated about 15m far from the rest of the burials, was not included because its stratigraphic position with MP2 or MP3 cannot be defined secured (**Table H). Results of the Bayesian outlier analysis for Ban Chiang.** Prior probabilities are the outlier probabilities set before the model run, whilst the posterior probabilities denote out outlying each determination in within the overall sequence. A posterior outlier probability of 50% means that that determination is left out of the model in half of the total run. The outlier models used are shown in the table as well. The prior outlier probability for most determinations in the model was set at 0.05. The table lists the prior and posterior outlier results and well as the type of model used (see Bronk Ramsey 2009), it can be seen that there are only two outliers of significance (OxA-30646 and OxA-22378) which are 100% outliers and therefore not included in the modelling runs. There is a further date (OxA-X-2436–53) which is 62% likely to be an outlier and a final determination (OxA-30671) that is 40% likely outlying. The combined data (duplicate dates of the same burial) are shown in italic. In asterisk are the dates from the 1974 excavation, all others are from the 1975 season (**Table I). Outlier detection results from the site of Non Nok Tha.** See caption for S1 file table I for details (**Table J). Outlier detection results from the site of Ban Lum Khao.** See caption for S1 file table J for details **(Table K). Outliers from the Ban Na Di model.** See caption to S1 file Table I for details (**Table L). %N measurements of bone from the Non Nok Tha site.** Anything below 0.8–1% is very unlikely to contain intact collagen enough for a radiocarbon determination. Human bones from burial contexts indicated with an asterisk (*) underwent collagen extraction but either no collagen was found or not enough for a radiocarbon determination (**Table M).**
(DOCX)
