# Correction: A New Chronology for the Bronze Age of Northeastern Thailand and Its Implications for Southeast Asian Prehistory

**DOI:** 10.1371/journal.pone.0142511

**Published:** 2015-11-04

**Authors:** The PLOS ONE Staff

There is an error in the third paragraph of the Acknowledgments. It should read: Ban Na Di: The site was excavated in 1981 by CFWH and Dr Amphan Kijngam, and we thank the New Zealand University Grants Committee and the Ford Foundation, for financial support.

S1 File was mistakenly published with tracked changes. The publisher apologizes for this error. Please see the correct version of S1 File here.

## Supporting Information

S1 File Supplementary Tables. Radiocarbon determinations from the Ban Chiang site. Asterisked samples (*) in the context column means material from the 1974 excavation season. The rest of the samples come from the 1975 excavation season. OxA-X- prefixes are given in preference to OxA- numbers when there is a problem with the pre-treatment chemistry, AMS measurement or when there is a novel or experimental protocol applied in the dating. Samples marked with an “S” (^**s**^) are those given a solvent extraction prior to collagen preparation to remove glues or conservatives identified on the bones. Date in this table stands for the conventional radiocarbon age, expressed in years BP. Errors are the determined standard errors (values are ± one standard error). ‘Used’ represents the amount of bone powder pretreated in milligrams. Yield represents the wSeight of collagen or ultrafiltered collagen in milligrams. Yield (%) is the percent yield of extracted collagen as a function of the starting weight of the bone analysed. %C is the carbon present in the combusted collagen. Stable isotope ratios are expressed in ‰ relative to vPDB with a mass spectrometric precision of ±0.2‰ for C and ±0.3‰ for N. C:N is the atomic ratio of C to N and is acceptable if it ranges between 2.9–3.5. ¶ denotes duplicate measurements on the same bone **(Table A). Radiocarbon determinations of human bone from the Non Nok Tha site**. See caption for SI Table for details. ^∫^denotes samples with low collagen yields for which ultrafiltration was not possible **(Table B). Radiocarbon dates from Ban Lum Khao**. Due to the bad preservation of bone collagen from the site, shell and charcoal samples was dated instead of bone. Burial 52 was dated at two labs (Oxford and Waikato) and both determinations exhibit good agreement. Wk-40470 is much older than OxA-29141, both from Bronze Age burial 89; the age of the former shell however is identical to the ages obtained from the Neolithic occupation, hence it is most likely part of the burial infill (disturbed sediment which include material from the lower Neolithic layers) rather than the grave goods. Note that the Neolithic charcoal determinations have much larger standard errors as they were produced using conventional methodologies **(Table C). Radiocarbon AMS dates of human bone from Ban Na Di**. See S1 File table A caption for details. ^∫^ denotes samples treated until the gelatinization step and not ultrafiltration was applied due to low collagen yield. ¶ denotes autoduplicate dates, i.e. repeat dates of the same bone **(Table D). Results of the Bayesian modelling of the Ban Chiang sequence**. Bold titles show the names of the successive phases. Italic scripts denote the calculated boundaries. Radiocarbon likelihoods (simple calibrated ages) are shown in the ‘Unmodelled’ columns, the ‘Modelled’ column shows the posterior probability ranges for each part of the main model. Convergence values are also shown **(Table E). Results of the Bayesian modelling from the site of Non Nok Tha**. See S1 File table A caption for details of the values in this table **(Table F). Results of the calibration and the Bayesian modelling from the site of Ban Lum Khao**. See Caption for S1 File table E for details of the values in this table **(Table G)**. **Results of the Bayesian modelling of the Ban Na Di site, Area A (bottom) and Area B (top)**. See caption to S1 File table E for details. Burial 15, excavated about 15m far from the rest of the burials, was not included because its stratigraphic position with MP2 or MP3 cannot be defined secured **(Table H). Results of the Bayesian outlier analysis for Ban Chiang**. Prior probabilities are the outlier probabilities set before the model run, whilst the posterior probabilities denote out outlying each determination in within the overall sequence. A posterior outlier probability of 50% means that that determination is left out of the model in half of the total run. The outlier models used are shown in the table as well. The prior outlier probability for most determinations in the model was set at 0.05. The table lists the prior and posterior outlier results and well as the type of model used (see Bronk Ramsey 2009), it can be seen that there are only two outliers of significance (OxA-30646 and OxA-22378) which are 100% outliers and therefore not included in the modelling runs. There is a further date (OxA-X-2436–53) which is 62% likely to be an outlier and a final determination (OxA-30671) that is 40% likely outlying. The combined data (duplicate dates of the same burial) are shown in italic. In asterisk are the dates from the 1974 excavation, all others are from the 1975 season **(Table I). Outlier detection results from the site of Non Nok Tha**. See caption for S1 File table I for details **(Table J). Outlier detection results from the site of Ban Lum Khao**. See caption for S1 File table J for details **(Table K). Outliers from the Ban Na Di model**. See caption to S1 File Table I for details **(Table L). %N measurements of bone from the Non Nok Tha site**. Anything below 0.8–1% is very unlikely to contain intact collagen enough for a radiocarbon determination. Human bones from burial contexts indicated with an asterisk (*) underwent collagen extraction but either no collagen was found or not enough for a radiocarbon determination **(Table M)**.
(DOCX)
