# Supplementary figures and images for: A New Chronology for the Bronze Age of Northeastern Thailand and Its Implications for Southeast Asian Prehistory
Source: PLoS One. 2015 Sep 18;10(9):e0137542. doi: 10.1371/journal.pone.0137542 (PMC4575132; doi:10.1371/journal.pone.0137542)

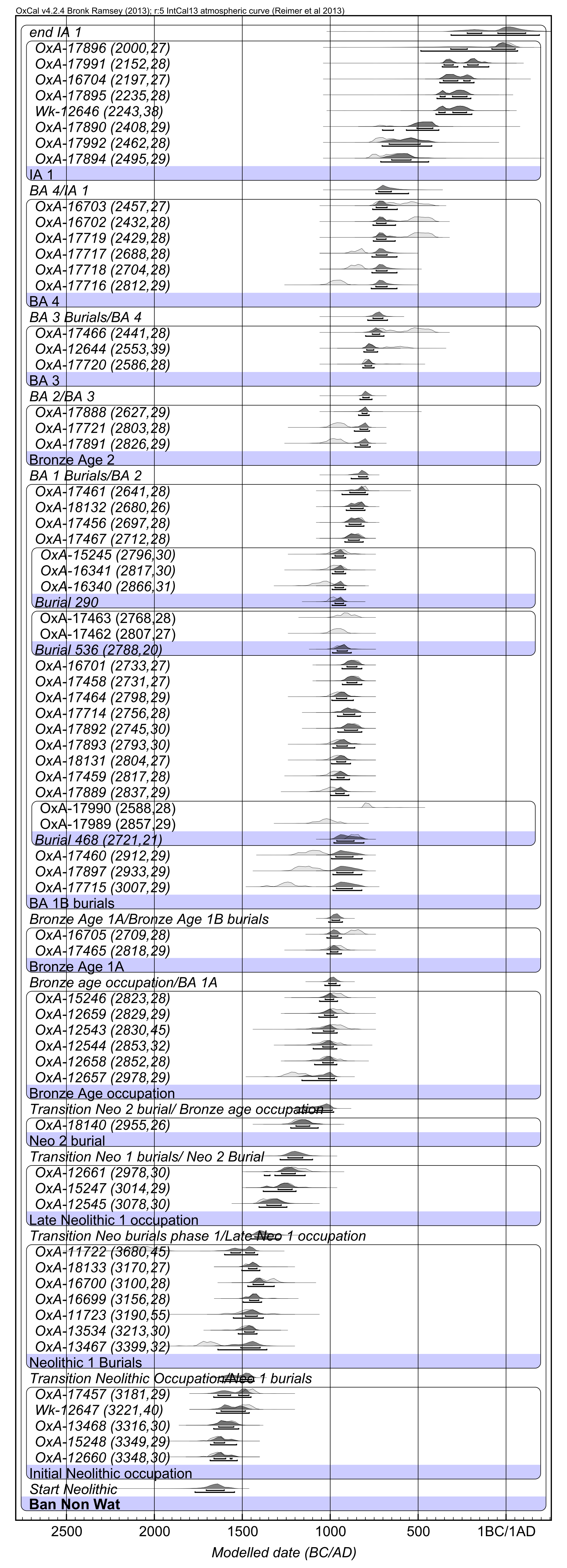

Supplement: S1 Fig — (OxCal. v4.2.4 Bronk Ramsey (2009 [38]); r:5 IntCal13 atmospheric curve (Reimer et al. 2013 [39]). (TIFF) [file pone.0137542.s001.tiff]

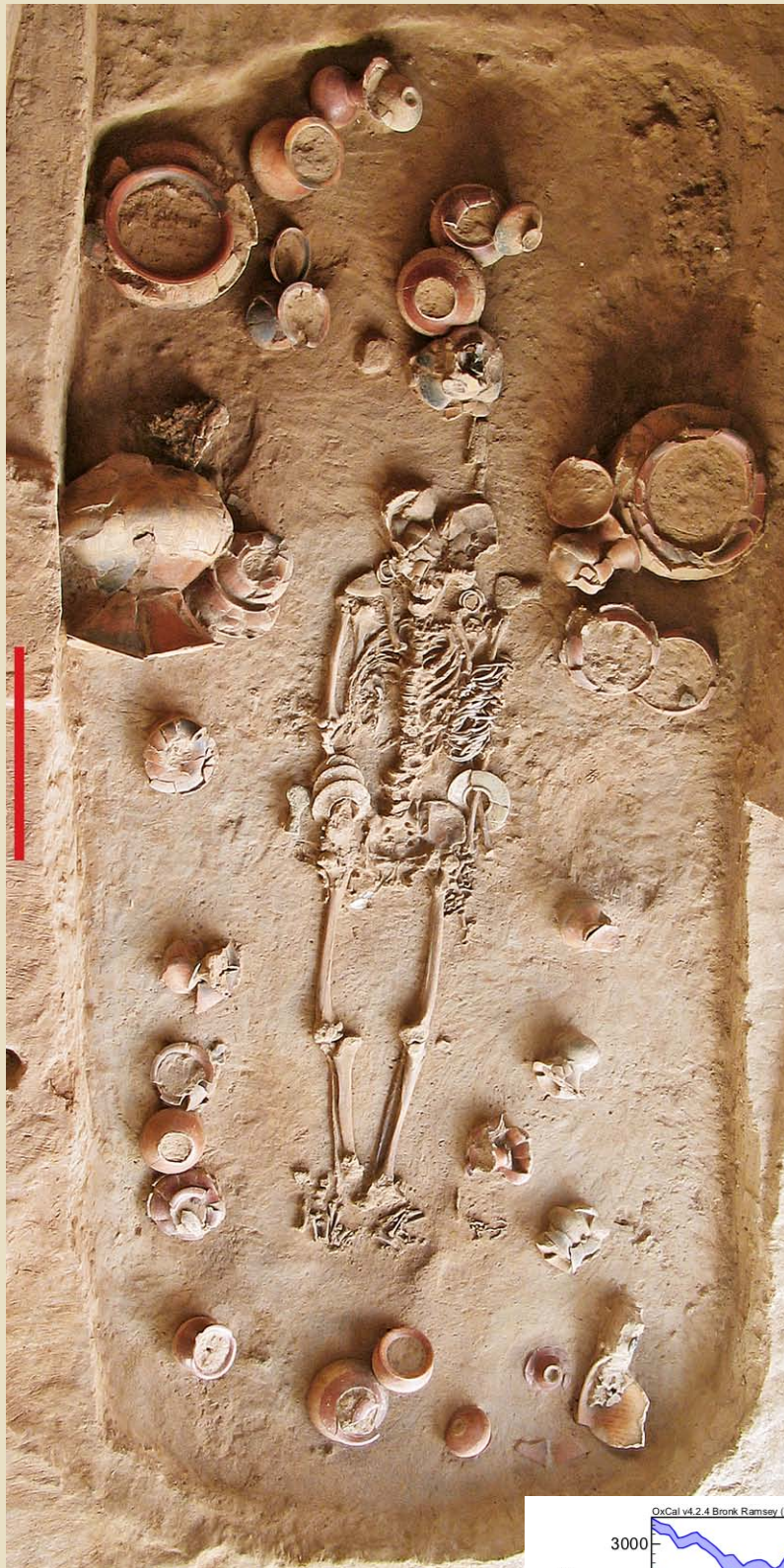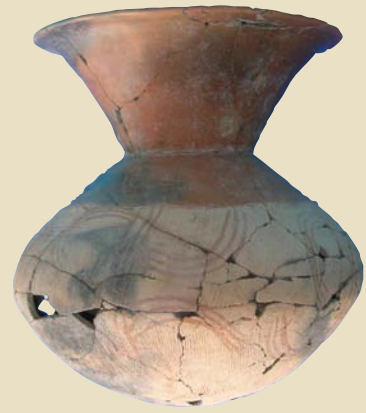

20 cm

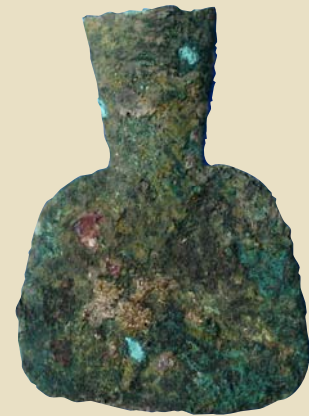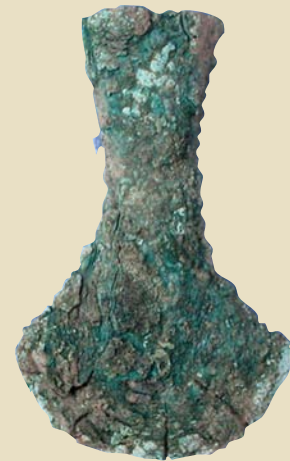

5 cm

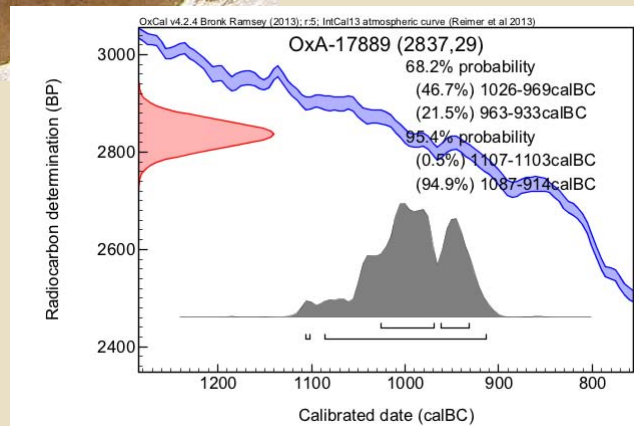

Supplement: S4 Fig — Male individual interred with multiple copper artefacts including socketed axes. The ceramic vessel is decorated with a frieze of dancers. The calibrated AMS determinations for this burial is 1110–915 BC. (PDF) [file pone.0137542.s004.pdf]

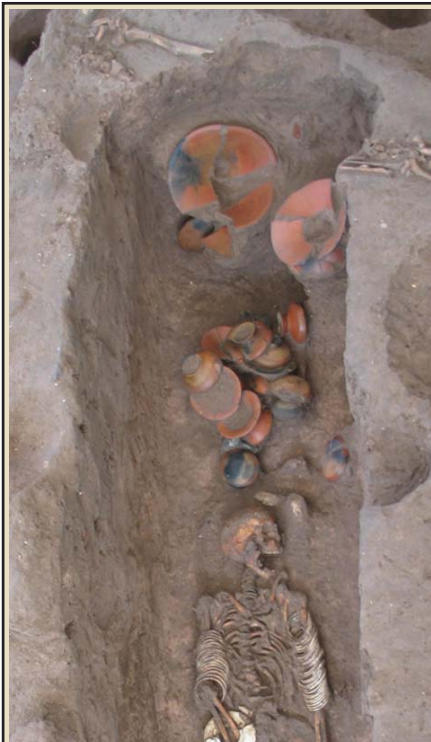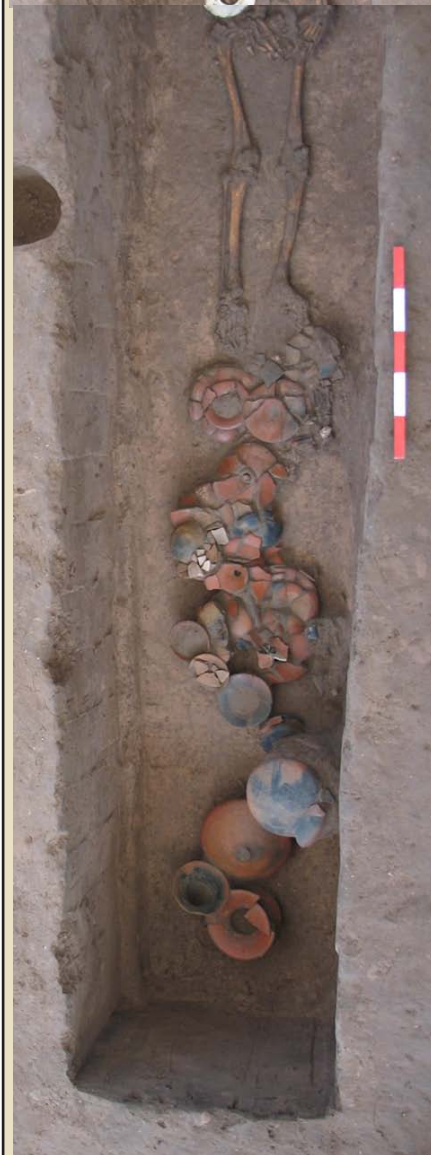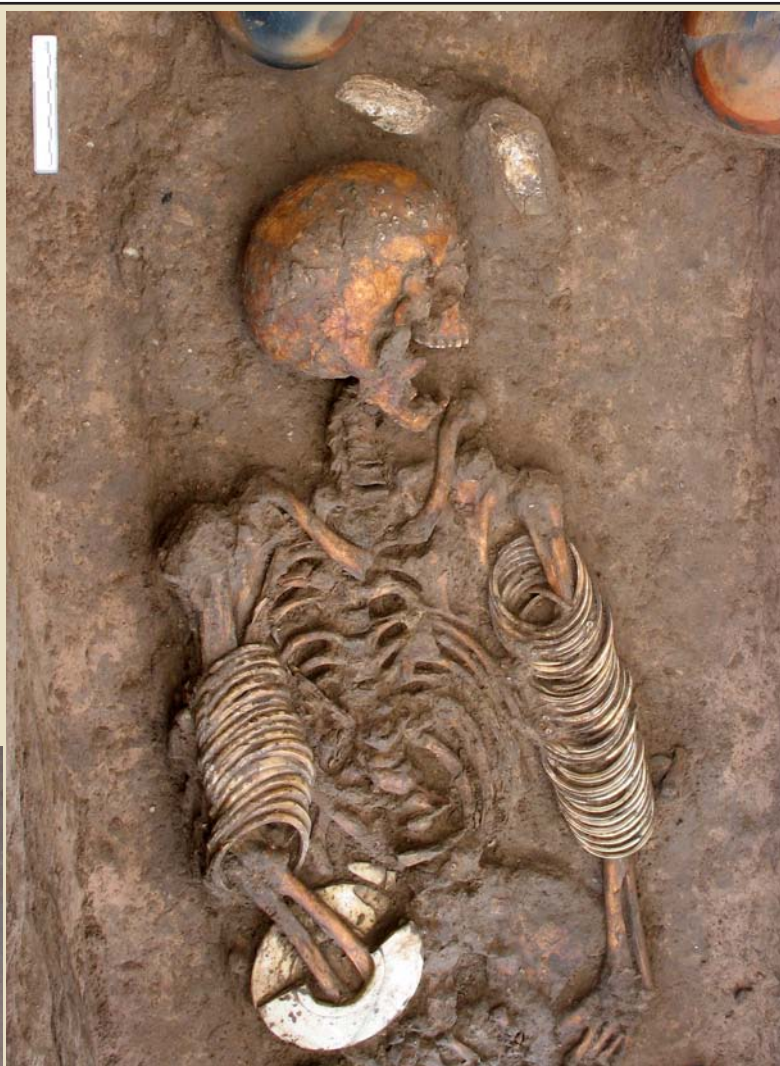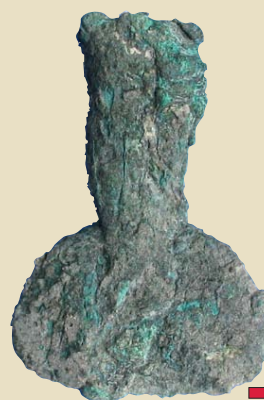

5 cm

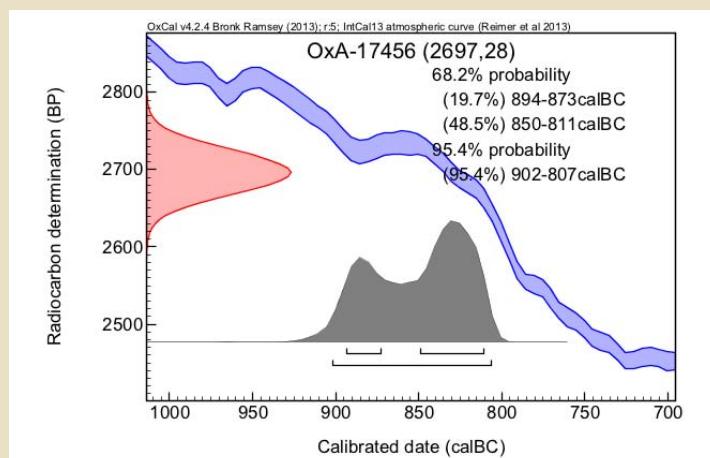

Supplement: S5 Fig — Male individual interred with a copper axe and many ceramic vessels. The dated bivalve shell lies by the head, and the calibrated radiocarbon age is 900–805 BC. (PDF) [file pone.0137542.s005.pdf]

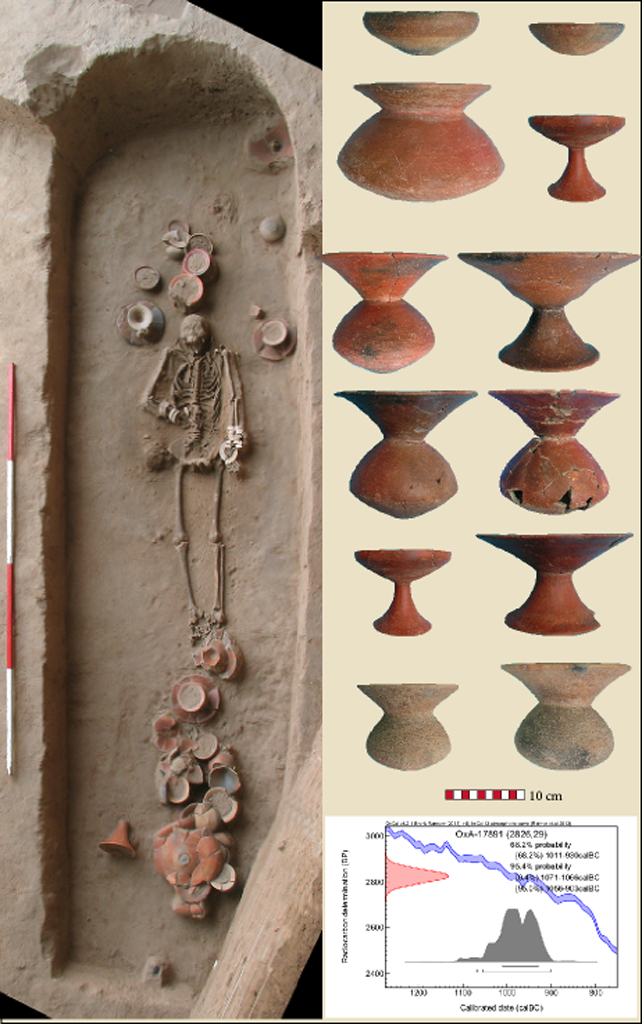

Supplement: S6 Fig — Female individual interred with many pottery vessels thought to reflect lavish mortuary feasting. The calibrated radiocarbon age for this burial is 1075–900 BC. (TIF) [file pone.0137542.s006.tif]

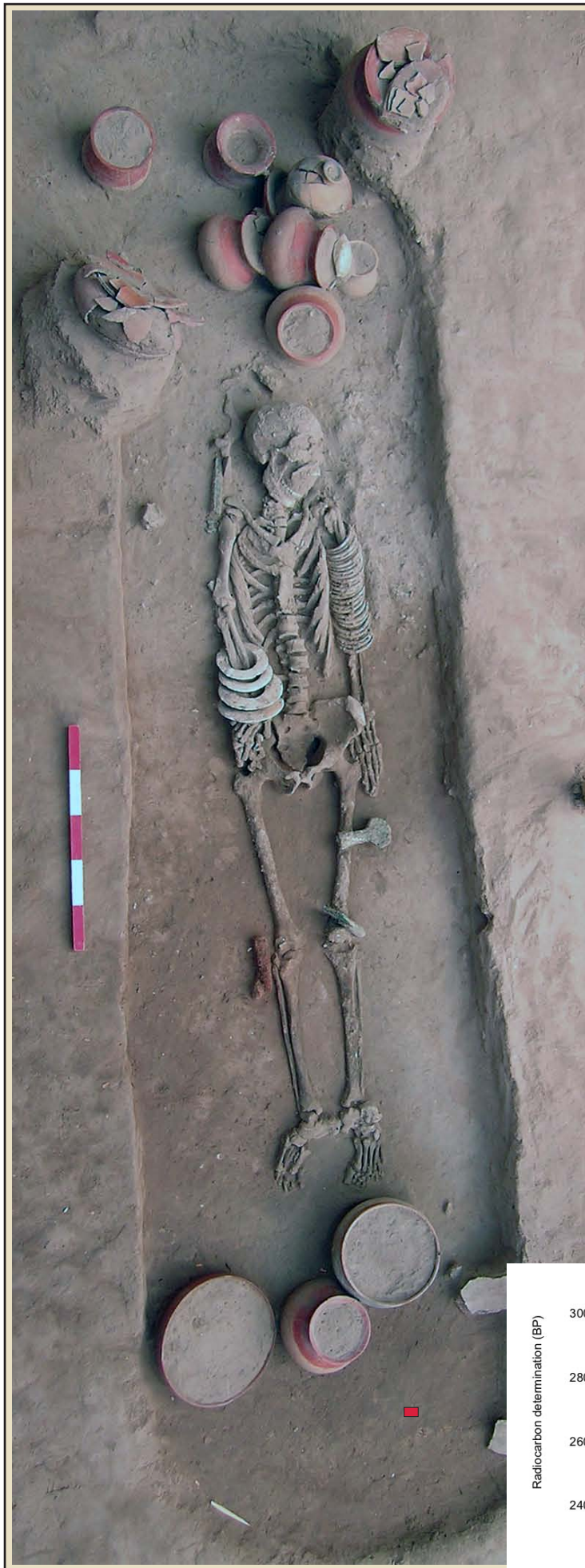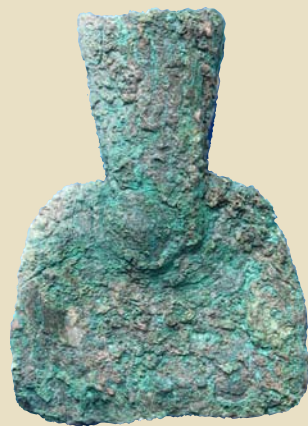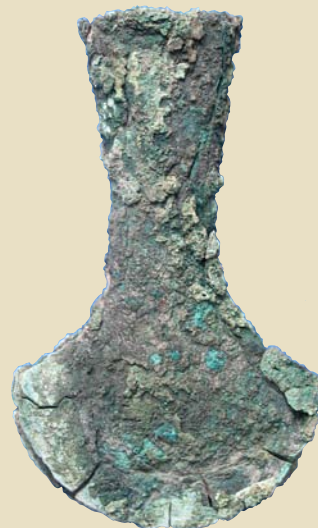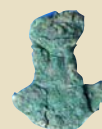

5 cm

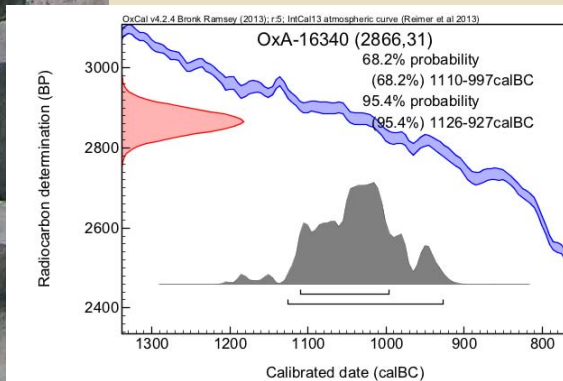

Supplement: S7 Fig — Male individual interred with three copper socketed axes and a copper chisel. The calibrated radiocarbon age for this burial is 1125–930 BC. (PDF) [file pone.0137542.s007.pdf]

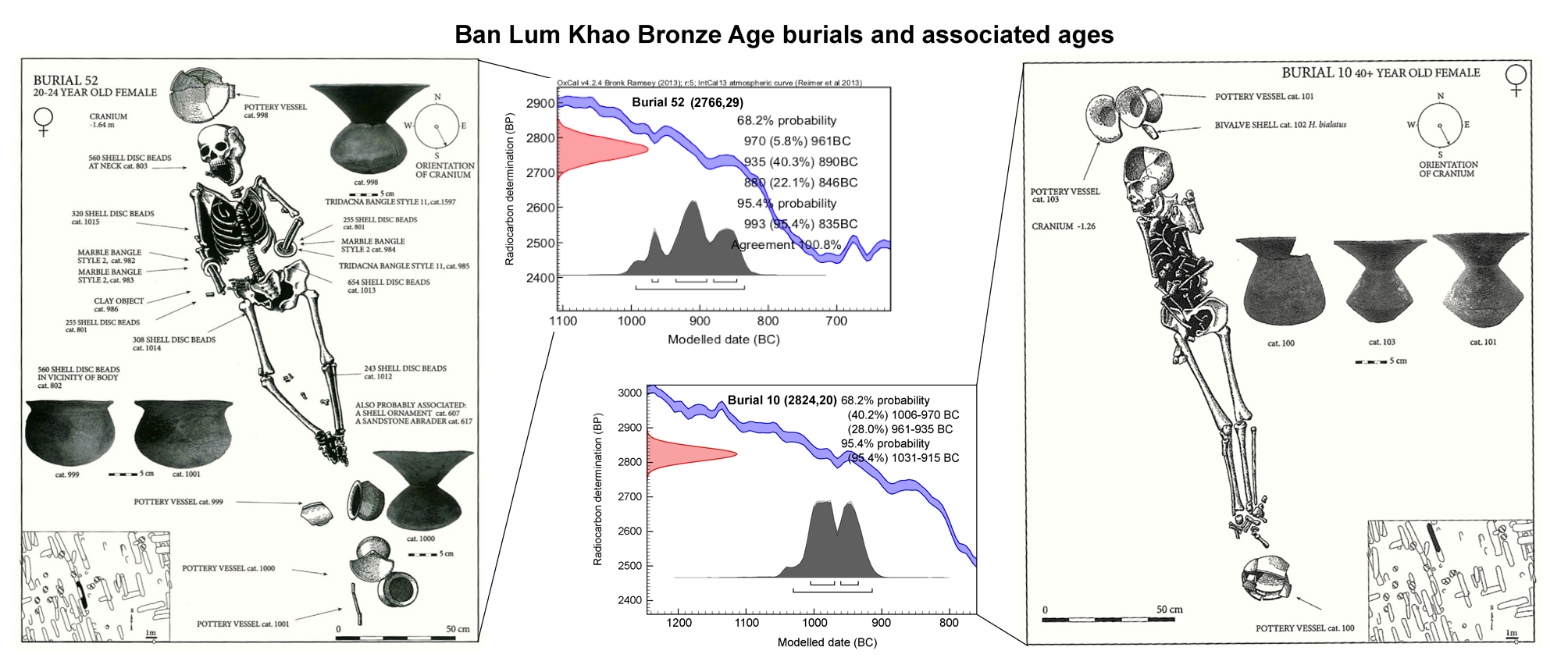

Supplement: S8 Fig — The mortuary offerings are much poorer than those at contemporary Ban Non Wat. (TIF) [file pone.0137542.s008.tif]
