# Supplementary material for: A New Chronology for the Bronze Age of Northeastern Thailand and Its Implications for Southeast Asian Prehistory
Source: PLoS One. 2015 Sep 18;10(9):e0137542. doi: 10.1371/journal.pone.0137542 (PMC4575132; doi:10.1371/journal.pone.0137542)
Supplement: S1 File — Radiocarbon determinations from the Ban Chiang site. Asterisked samples (*) in the context column means material from the 1974 excavation season. The rest of the samples come from the 1975 excavation season. OxA-X- prefixes are given in preference to OxA- numbers when there is a problem with the pre-treatment chemistry, AMS measurement or when there is a novel or experimental protocol applied in the dating. Samples marked with an “S” (s) are those given a solvent extraction prior to collagen preparation to remove glues or conservatives identified on the bones. Date in this table stands for the conventional radiocarbon age, expressed in years BP. Errors are the determined standard errors (values are ± one standard error). ‘Used’ represents the amount of bone powder pretreated in milligrams. Yield represents the wSeight of collagen or ultrafiltered collagen in milligrams. Yield (%) is the percent yield of extracted collagen as a function of the starting weight of the bone analysed. %C is the carbon present in the combusted collagen. Stable isotope ratios are expressed in ‰ relative to vPDB with a mass spectrometric precision of ±0.2‰ for C and ±0.3‰ for N. C:N is the atomic ratio of C to N and is acceptable if it ranges between 2.9–3.5. ¶ denotes duplicate measurements on the same bone (Table A). Radiocarbon determinations of human bone from the Non Nok Tha site. See caption for SI Table for details. ∫denotes samples with low collagen yields for which ultrafiltration was not possible (Table B). Radiocarbon dates from Ban Lum Khao. Due to the bad preservation of bone collagen from the site, shell and charcoal samples was dated instead of bone. Burial 52 was dated at two labs (Oxford and Waikato) and both determinations exhibit good agreement. Wk-40470 is much older than OxA-29141, both from Bronze Age burial 89; the age of the former shell however is identical to the ages obtained from the Neolithic occupation, hence it is most likely part of the burial infill (disturb [file pone.0137542.s009.docx]

**Supporting information**

**A new chronological framework for the Bronze Age of Southeast Asia**

*Oxford Laboratory methods for the dating of bone samples*

The ORAU undertook radiocarbon dating of bone and a small number of shell samples too. Treatment was undertaken using the methods outlined by Brock *et al*. [22] and also in the main text of the present article. For a small number of samples ultrafiltration was not performed because of the very low collagen yield after the gelatisation step.

Combusted gelatin samples were analysed using an EA-CF-IRMS system (SERCON 20-22 IRMS linked with a SERCON GSL elemental analyser with a He carrier gas operating in continuous flow mode. This approach enables the measurement of δ^15^N and δ^13^C, nitrogen and carbon content and calculation of C:N atomic ratios. Nylon and alanine were used as internal reference materials for calculating the stable isotope values. δ^13^C values for radiocarbon measurements cited in this section are reported with reference to VPDB [40]. The CO_2_ was cryogenically distilled and then graphitized using published methods, before AMS dating.

Radiocarbon dates of bone are reported in S1-S4 Tables. Bones were screened before being passed to collagen extraction by measuring %Nitrogen. Many bones were poorly preserved in terms of collagen, with several <than 1% wt. collagen (the effective threshold in the ORAU). Analytical parameters we measured, however, principally including the carbon to nitrogen atomic ratio and the %C of the collagen, were well within acceptable ranges. In addition, only one of the four significant outliers in the Ban Chiang model was of a low collagen yield, the remainder fitted perfectly within the Bayesian model, suggesting no significant age differences can be associated with low collagen yielding bone in this site. The same applies to the determinations of bone from the sites of Ban Na Di and Non Nok Tha. Duplicate measurements, undertaken from the start of the pretreatment chemistry, also showed good statistical agreement, supporting the accuracy of the radiocarbon measurements we have produced.

*Waikato Laboratory*

A series of shells and charcoal samples from Ban Lum Khao were dated at the Waikato Radiocarbon Laboratory, New Zealand. The methods and calculation of dates are very similar to the Oxford routine methods and will not be repeated here. See also main text for a description.

*Bayesian modelling:*

All dated samples have been included in Bayesian chronometric models. These models are based on the stratigraphic sequence information described in the main paper and in individual site reports. We used OxCal 4.2.2 [38] and the INTCAL13 calibration curve [39] in the Bayesian modelling. We ran the models multiple times at several million iterations to assess reproducibility. The Bayesian method enables the archaeological stratigraphic information to be incorporated in the chronometric modelling, along with the radiocarbon likelihoods. The model framework reflects the series of phases and archaeological strata excavated through the sequence of the sites. Start and end boundaries bracket each of the archaeological phases. Where there is a sterile layer, two boundaries are included. We generate probability distribution functions (PDF) for the beginning and ending of each phase and this summary data is shown in the main paper (Figure 8), where each of the principal sites are compared precisely where the transition to the Bronze Age phases lie in the chronometric model.

Each site model was reproducible with high convergence values, consistently above 99.0%. This shows that the algorithms are able to converge or calculate the posterior probabilities quickly. We use outlier detection analysis to downweigh individual determinations that are in disagreement with the overall prior framework of the model. General, SSimple, Charcoal outlier models were used [41]. The data from each model are shown in the following SI Tables 5-12.

40. Coplen TB (1994) Reporting of stable hydrogen, carbon, and oxygen isotopic abundances. Pure and Applied Chemistry 66(2), 273–6.

41. Bronk Ramsey C (2009a) Dealing with outliers and offsets in radiocarbon dating, Radiocarbon 51(3), 1023-1045.

S1 File. Supplementary Tables

**S1 Table A. Radiocarbon determinations from the Ban Chiang site.** Asterisked samples (*) in the context column means material from the 1974 excavation season. The rest of the samples come from the 1975 excavation season. OxA-X- prefixes are given in preference to OxA- numbers when there is a problem with the pre-treatment chemistry, AMS measurement or when there is a novel or experimental protocol applied in the dating. Samples marked with an “S” (**^s^**) are those given a solvent extraction prior to collagen preparation to remove glues or conservatives identified on the bones. Date in this table stands for the conventional radiocarbon age, expressed in years BP [38]. Errors are the determined standard errors (values are ± one standard error). ‘Used’ represents the amount of bone powder pretreated in milligrams. Yield represents the weight of collagen or ultrafiltered collagen in milligrams. Yield (%) is the percent yield of extracted collagen as a function of the starting weight of the bone analysed. %C is the carbon present in the combusted collagen. Stable isotope ratios are expressed in ‰ relative to vPDB with a mass spectrometric precision of ±0.2‰ for C and ±0.3‰ for N. C:N is the atomic ratio of C to N and is acceptable if it ranges between 2.9-3.5. ¶ denotes duplicate measurements on the same bone.

| **OxA-** | **Radiocarbon age BP** | **±** | **Context** | **Material** | **Used (mg)** | **Collagen Yield (mg)** | **%Yld** | **%C** | **δ^13^C (‰)** | **δ^15^N (‰)** | **CN** |
| --- | --- | --- | --- | --- | --- | --- | --- | --- | --- | --- | --- |
| 22378 | 2965 | 29 | Burial 29 | Bone *(Sus scrofa)* | 1250 | 16 | 1.3 | 44.7 | -20.9 | 8.8 | 3.4 |
| X-2559-13¶ | 2584 | 23 | Burial 56 | Bone *(Sus scrofa)* | 700 | 22.3 | 3.2 | 42.1 | -19.5 | 8.1 | 3.4 |
| 22380¶ | 2516 | 26 | Burial 56 | Bone *(Sus scrofa)* | 1210 | 8.87 | 0.7 | 44.5 | -19.4 | 8.7 | 3.2 |
| 22381 | 2786 | 26 | Burial 47 | Bone *(Sus scrofa)* | 1070 | 5.5 | 0.5 | 44.8 | -19.1 | 7.8 | 3.1 |
| 22383 | 2819 | 26 | Burial 54 | Bone *(Sus scrofa)* | 1450 | 23.9 | 1.6 | 45.2 | -19.6 | 7.9 | 3.1 |
| 24047 | 2868 | 26 | Burial 76 | Human bone | 620 | 6.07 | 1.0 | 43.2 | -18.3 | 10.6 | 3.2 |
| X-2442-24 | 3063 | 33 | Burial 33 * | Human bone | 1050 | 3.12 | 0.3 | 45.3 | -18.6 | 9.8 | 3.3 |
| 30671 | 2792 | 30 | Burial 33 * | Human bone | 1060 | 31.54 | 3 | 40.9 | -18.8 | 9.1 | 3.3 |
| 25014 | 2984 | 26 | Burial 43 * | Human bone | 960 | 8.5 | 0.9 | 44.6 | -18.3 | 11.1 | 3.3 |
| 25015**^s^** | 3242 | 26 | Burial 44 * | Human bone | 950 | 11.11 | 1.2 | 45.7 | -18.3 | 10.5 | 3.2 |
| X-2438-16 | 2958 | 29 | Burial 45 * | Human bone | 2200 | 2.11 | 0.1 | 41.1 | -18.5 | 10.3 | 3.3 |
| X-2438-17**^s^** | 2978 | 31 | Burial 47 * | Human bone | 1020 | 2.5 | 0.2 | 43.1 | -18.9 | 11.0 | 3.3 |
| X-2436-53 | 2936 | 25 | Burial 47 | Human bone | 1260 | 4.15 | 0.3 | 42.9 | -18.0 | 10.6 | 3.3 |
| 25016¶ | 2789 | 26 | Burial 49 | Human bone | 2130 | 42.5 | 2 | 41.6 | -18.3 | 10.9 | 3.2 |
| 25017¶ | 2801 | 25 | Burial 49 | Human bone | 650 | 20.4 | 3.1 | 43.4 | -18.3 | 10.7 | 3.2 |
| 25018 | 2844 | 26 | Burial 65 | Human bone | 1170 | 19.5 | 1.7 | 44.2 | -18.9 | 10.9 | 3.2 |
| 25019 | 2810 | 25 | Burial 72 | Human bone | 1190 | 20.33 | 1.7 | 42.4 | -18.4 | 10.5 | 3.2 |
| 30362 | 2277 | 25 | Burial 11 * | Human bone | 1020 | 19.74 | 1.9 | 41.9 | -19.1 | 10.4 | 3.2 |
| X-2583-28 | 2628 | 24 | Burial 12 * | Human bone | 1010 | 6.4 | 0.6 | 40.8 | -18.7 | 11.4 | 3.2 |
| X-2583-34 | 2511 | 25 | Burial 21 * | Human bone | 1060 | 4.21 | 0.4 | 40.2 | -18.9 | 10.6 | 3.3 |
| 30397 | 2842 | 29 | Burial 31 * | Human bone | 1030 | 10.47 | 1 | 40.7 | -19.1 | 10.0 | 3.2 |
| X-2583-35 | 2577 | 26 | Burial 39 * | Human bone | 1040 | 6.33 | 0.6 | 41.4 | -18.4 | 10.8 | 3.2 |
| 30363 | 2976 | 25 | Burial 41 * | Human bone | 1000 | 20.19 | 2 | 40.6 | -18.5 | 9.8 | 3.2 |
| 30433 | 1886 | 26 | Burial 6 | Human bone | 1100 | 19.4 | 1.8 | 42.5 | -18.6 | 10.3 | 3.3 |
| 30434**^s^** | 1864 | 27 | Burial 9 | Human bone | 1080 | 21.24 | 2 | 42.3 | -18.9 | 10.2 | 3.3 |
| 30435**^s^** | 2245 | 27 | Burial 19 | Human bone | 1080 | 18.48 | 1.7 | 41 | -19.1 | 10.5 | 3.3 |
| 30645 | 2232 | 30 | Burial 20 | Human bone | 1030 | 40.18 | 3.9 | 43 | -18.6 | 10.7 | 3.3 |
| 30646 | 2931 | 30 | Burial 23 | Human bone | 1020 | 11.23 | 1.1 | 38.8 | -18.1 | 10.0 | 3.4 |
| 30647 | 2202 | 29 | Burial 24 | Human bone | 1030 | 44.74 | 4.3 | 43.4 | -18.1 | 10.9 | 3.3 |
| 30648 | 2498 | 29 | Burial 25 | Human bone | 1100 | 32.5 | 3 | 43.2 | -17.9 | 10.8 | 3.2 |
| 30649 | 2499 | 30 | Burial 27 | Human bone | 1000 | 25.76 | 2.6 | 43.7 | -18.8 | 10.7 | 3.2 |
| 30650**^s^** | 2553 | 29 | Burial 29 | Human bone | 1040 | 44.1 | 4.2 | 43.5 | -19.3 | 10.4 | 3.1 |
| 30651**^s^** | 2558 | 29 | Burial 30 | Human bone | 1050 | 37.37 | 3.6 | 42.1 | -19.5 | 10.9 | 3.1 |
| 30652 | 2774 | 29 | Burial 31 | Human bone | 1020 | 20.6 | 2 | 42.7 | -17.7 | 9.9 | 3.1 |
| 30653 | 2632 | 29 | Burial 33 | Human bone | 1010 | 23.81 | 2.4 | 43.2 | -18.4 | 9.2 | 3.1 |
| 30654 | 2690 | 29 | Burial 34 | Human bone | 990 | 32.23 | 3.3 | 42.9 | -18.9 | 9.0 | 3.1 |
| 30655 | 2767 | 30 | Burial 35 | Human bone | 1020 | 22.24 | 2.2 | 42.5 | -18.3 | 9.4 | 3.1 |
| 30656 | 2469 | 29 | Burial 36 | Human bone | 990 | 20 | 2 | 40.8 | -18.2 | 10.7 | 3.3 |
| 30657**^s^** | 2327 | 30 | Burial 40 | Human bone | 990 | 27.45 | 2.8 | 41 | -18.5 | 10.7 | 3.2 |
| 30658 | 2307 | 29 | Burial 41 | Human bone | 1060 | 41.37 | 3.9 | 40.5 | -19.1 | 10.5 | 3.2 |
| 30659¶ | 2632 | 30 | Burial 42 | Human bone | 1030 | 37.68 | 3.7 | 40.7 | -18.6 | 9.6 | 3.3 |
| 30660¶ | 2637 | 29 | Burial 42 | Human bone | 1020 | 31.63 | 3.1 | 40.4 | -18.4 | 10.0 | 3.3 |
| 30661**^s^** | 2850 | 30 | Burial 45 | Human bone | 1110 | 18.89 | 1.7 | 40.3 | -18.4 | 10.1 | 3.3 |
| 30662 | 2782 | 30 | Burial 46 | Human bone | 990 | 25.32 | 2.6 | 40.7 | -18.7 | 10.0 | 3.3 |
| 30663**^s^** | 2753 | 30 | Burial 51 | Human bone | 1010 | 17.01 | 1.7 | 40.5 | -18.3 | 10.8 | 3.3 |
| 30664 | 2518 | 30 | Burial 53 | Human bone | 980 | 6.87 | 0.7 | 41.1 | -18.3 | 10.2 | 3.3 |
| 30665**^s^** | 2815 | 31 | Burial 55 | Human bone | 1010 | 19.3 | 1.9 | 41.5 | -18.5 | 10.2 | 3.3 |
| X-2590-19 | 2513 | 30 | Burial 56 | Human bone | 1060 | 3.76 | 0.4 | 42.8 | -18.2 | 11.7 | 3.4 |
| 30666 | 2568 | 29 | Burial 59 | Human bone | 1100 | 47.51 | 4.3 | 41.2 | -18.5 | 10.5 | 3.3 |
| 30667 | 2701 | 30 | Burial 61 | Human bone | 1070 | 22.72 | 2.1 | 41 | -18.7 | 9.7 | 3.3 |
| 30668 | 2409 | 32 | Burial 73 | Human bone | 1050 | 43.84 | 4.2 | 41.2 | -18.1 | 10.1 | 3.3 |
| 30669 | 2793 | 33 | Burial 76 | Human bone | 1040 | 13.68 | 1.3 | 41.7 | -18.2 | 10.4 | 3.4 |
| 30670 | 2349 | 30 | Burial 78 | Human bone | 1040 | 19.64 | 1.9 | 40.8 | -18.2 | 10.3 | 3.3 |
| X-2593-43 | 3061 | 30 | Burial 74 | Human bone | 1070 | 4.97 | 0.5 | 41.2 | -18.8 | 10.3 | 3.4 |

**S1 file Table B: Radiocarbon determinations of human bone from the Non Nok Tha site.** See caption for S1 file table A for details. ^∫^ denotes samples with low collagen yields for which ultrafiltration was not possible.

| **OxA-** | **Radiocarbon age BP** | **±** | **Context** | **Material** | **Used (mg)** | **Collagen Yield (mg)** | **%Yld** | **%C** | **δ^13^C (‰)** | **δ^15^N (‰)** | **CN** |
| --- | --- | --- | --- | --- | --- | --- | --- | --- | --- | --- | --- |
| X-2586-15 | 2570 | 27 | Burial 85 | Human bone | 1020 | 3.72 | 0.4 | 42.3 | -19.0 | 12.3 | 3.4 |
| X-2586-16 | 2505 | 31 | Burial 80 | Human bone | 1040 | 3.58 | 0.3 | 40.8 | -18.3 | 12.1 | 3.4 |
| X-2586-17 | 3146 | 30 | Burial 94 | Human bone | 960 | 2.61 | 0.3 | 43 | -19.5 | 14.8 | 3.4 |
| X-2586-18 | 3102 | 30 | Burial 94 | Human bone | 1010 | 3.07 | 0.3 | 39.7 | -19.5 | 13.0 | 3.5 |
| X-2586-19 | 2564 | 30 | Burial 1 | Human bone | 910 | 3.36 | 0.4 | 41.1 | -19.5 | 14.3 | 3.4 |
| X-2524-20 ^∫^ | 2669 | 27 | Burial 38 | Human bone | 1530 | 19.02 | 1.2 | 26.1 | -11.5 | 6.6 | 3.4 |
| X-2524-21^∫^ | 2717 | 25 | Burial 62 | Human bone | 1610 | 20.78 | 1.3 | 25.9 | -19.5 | 10.7 | 3.4 |
| 30360 | 2911 | 27 | Burial 121 | Human bone | 1140 | 14.9 | 1.3 | 41.9 | -18.9 | 12.7 | 3.2 |
| 30361 | 2879 | 26 | Burial 78 | Human bone | 1100 | 9.81 | 0.9 | 41.3 | -19.1 | 11.4 | 3.2 |
| 30391 | 2414 | 27 | Burial 24 | Human bone | 1020 | 6.09 | 0.6 | 40.4 | -19.3 | 11.0 | 3.3 |
| 30392 | 3059 | 28 | Burial 29 | Human bone | 990 | 10.49 | 1.1 | 41.7 | -19.3 | 11.7 | 3.4 |
| 30393 | 2473 | 27 | Burial 7 | Human bone | 1040 | 10.06 | 1 | 41.1 | -18.9 | 11.5 | 3.3 |
| 30394 | 2566 | 27 | Burial 17 | Human bone | 1020 | 29.05 | 2.8 | 41.8 | -18.7 | 10.7 | 3.3 |
| 30395 | 2536 | 28 | Burial 55 | Human bone | 1030 | 8.07 | 0.8 | 40.5 | -19.2 | 10.3 | 3.3 |
| 30396 | 3028 | 28 | Burial 35 | Human bone | 1080 | 17.82 | 1.7 | 41.3 | -19.1 | 12.5 | 3.3 |
| 30644 | 2788 | 29 | Burial 79 | Human bone | 1000 | 10.28 | 1 | 41.9 | -19.2 | 10.8 | 3.3 |

**S1 file Table C: Radiocarbon dates from Ban Lum Khao.** Due to the bad preservation of bone collagen from the site, shell and charcoal samples was dated instead of bone. Burial 52 was dated at two labs (Oxford and Waikato) and both determinations exhibit good agreement. Wk-40470 is much older than OxA-29141, both from Bronze Age burial 89; the age of the former shell however is identical to the ages obtained from the Neolithic occupation, hence it is most likely part of the burial infill (disturbed sediment which include material from the lower Neolithic layers) rather than the grave goods. Note that the Neolithic charcoal determinations have much larger standard errors as they were produced using conventional methodologies.

| **OxA-** | **Radiocarbon age BP** | **±** | **Context** | **Material** | **Used (mg)** | **Yield (mg)** | **% C** | **δ^13^C (‰)** |
| --- | --- | --- | --- | --- | --- | --- | --- | --- |
| 29139 | 2640 | 27 | Burial 30 | Shell bead | 15.6 | 1.46 | 9.4 | -1.2 |
| 29140 | 2775 | 28 | Burial 59 | Shell bead | 32 | 3.3 | 10.3 | -12.4 |
| 29141 | 2753 | 27 | Burial 89 | *Pseudodon* sp. | 40 | 4 | 10 | -5.9 |
| 29142 | 2766 | 29 | Burial 52 | Shell bead | 38 | 3.3 | 8.7 | -4.4 |
| 29143 | 2608 | 26 | Burial 61 | Shell bead | 47 | 5 | 10.6 | -7.1 |
| 29144 | 2729 | 26 | Burial 62 | *Pseudodon* sp*.* | 27 | 2.2 | 8.1 | -7.0 |
| 29174 | 2748 | 27 | Burial 84 | Shell bead | 10.4 | 1 | 9.6 | -5.9 |
| 29175 | 2802 | 26 | Burial 107 | Shell bead | 9.58 | 1 | 10.4 | -6.6 |
| **Wk-** | **Radiocarbon age BP** | **±** | **Context** | **Material** | **Used (mg)** | **Yield (mg)** | **% C** | **δ^13^C (‰)** |
| 4507 | 3080 | 50 | Neolithic occupation | charcoal | n/a | n/a | n/a | n/a |
| 4508 | 3010 | 60 | Neolithic occupation | charcoal | n/a | n/a | n/a | n/a |
| 4509 | 3000 | 80 | Neolithic occupation | charcoal | n/a | n/a | n/a | n/a |
| 4510 | 3043 | 82 | Neolithic occupation | charcoal | n/a | n/a | n/a | n/a |
| 4511 | 3120 | 50 | Neolithic occupation | charcoal | n/a | n/a | n/a | n/a |
| 40468 | 2932 | 20 | Burial 70 | Worked shell | n/a | n/a | n/a | -1.6 |
| 40469 | 3014 | 20 | Burial 70 | Worked shell | n/a | n/a | n/a | -3.0 |
| 40464 | 2925 | 20 | Burial 28 | *Hyriopsis bialatus* | n/a | n/a | n/a | -10.1 |
| 40466 | 2950 | 20 | Burial 40 | *Cristaria plicata* | n/a | n/a | n/a | -1.8 |
| 40471 | 2918 | 20 | Burial 90 | *Hyriopsis bialatus* | n/a | n/a | n/a | -8.9 |
| 40465 | 2896 | 20 | Burial 29 | Not known | n/a | n/a | n/a | -7.7 |
| 40462 | 2824 | 20 | Burial 10 | *Hyriopsis bialatus* | n/a | n/a | n/a | -5.7 |
| 40467 | 2783 | 20 | Burial 52 | Shell bead | n/a | n/a | n/a | -4.5 |
| 40461 | 2762 | 20 | Burial 7 | *Hyriopsis bialatus* | n/a | n/a | n/a | -6.8 |
| 40470 | 3115 | 20 | Burial 89 | *Pseudodon* sp. | n/a | n/a | n/a | -5.1 |
| 40459 | 2720 | 20 | Burial 3 | *Hyriopsis bialatus* | n/a | n/a | n/a | -7.3 |
| 40463 | 2624 | 20 | Burial 27 | *Hyriopsis bialatus* | n/a | n/a | n/a | -7.5 |
| 40460 | 2564 | 20 | Burial 4 | *Hyriopsis bialatus* | n/a | n/a | n/a | -10.5 |

**S1 file Table D: Radiocarbon AMS dates of human bone from Ban Na Di.** See S1 file table A caption for details. ^∫^ denotes samples treated until the gelatinization step and not ultrafiltration was applied due to low collagen yield. ¶ denotes autoduplicate dates, i.e. repeat dates of the same bone.

| **OxA-** | **Radiocarbon age BP** | **±** | **Context** | **Material** | **Used (mg)** | **Collagen Yield (mg)** | **%Yld** | **%C** | **δ^13^C (‰)** | **δ^15^N (‰)** | **CN** |
| --- | --- | --- | --- | --- | --- | --- | --- | --- | --- | --- | --- |
| X-2524-27^∫^ | 2534 | 25 | Burial 39 | Human bone | 2040 | 7.6 | 0.4 | 28.3 | -18.4 | 10.0 | 3.5 |
| 28106^∫^ | 2497 | 27 | Burial 10 | Human bone | 1080 | 10.66 | 1 | 25.5 | -19.1 | 10.3 | 3.4 |
| 28125^∫^ | 2346 | 26 | Burial 15 | Human bone | 620 | 24.05 | 3.9 | 40.7 | -17.8 | 12.8 | 3.2 |
| 28126^∫^ | 2339 | 25 | Burial 9 | Human bone | 720 | 16.36 | 2.3 | 39.8 | -17.8 | 11.4 | 3.2 |
| 28430^∫^ | 2552 | 31 | Burial 42 | Human bone | 1650 | 6.57 | 0.4 | 44.8 | -18.3 | 11.2 | 3.5 |
| 30377¶ | 2594 | 24 | Burial 32 | Human bone | 1270 | 15.47 | 1.2 | 40.2 | -18.6 | 11.2 | 3.3 |
| 30414¶^∫^ | 2559 | 29 | Burial 32 | Human bone | 1270 | 22.38 | 1.8 | 32.5 | -18.8 | 11.9 | 3.4 |
| 30415 | 2515 | 27 | Burial 26 | Human bone | 1150 | 12 | 1 | 38.1 | -18.6 | 11.5 | 3.4 |
| 30416 | 2455 | 27 | Burial 29 | Human bone | 1150 | 12 | 1 | 37.8 | -18.5 | 11.1 | 3.4 |

**S1 file Table E: Results of the Bayesian modelling of the Ban Chiang sequence**. Bold titles show the names of the successive phases. Italic scripts denotes the boundaries. Radiocarbon likelihoods (simple calibrated ages) are shown in the ‘Unmodelled’ columns, the ‘Modelled’ column shows the posterior probability ranges for each part of the main model. Convergence values are also shown.

| **Ban Chiang** | **Unmodelled (BC/AD)** | | | | **Modelled (BC/AD)** | | | | **Convergence values** |
| --- | --- | --- | --- | --- | --- | --- | --- | --- | --- |
|  | **68.2% probability** | | **95.4% probability** | | **68.2%**  **probability** | | **95.4%**  **probability** | |  |
|  | ***from*** | ***to*** | ***from*** | ***to*** | ***from*** | ***to*** | ***from*** | ***to*** |  |
| *End LP and BC* | | | | | 105 | 310 | 80 | 665 | 98.8 |
| OxA-30434 Burial 9 (1864,27) | 85 | 215 | 75 | 225 | 80 | 170 | 70 | 225 | 99.9 |
| OxA-30433 Burial 6 (1886,26) | 70 | 135 | 60 | 215 | 75 | 135 | 60 | 215 | 100 |
| **LP Late Iron Age** | | | | |  | | | | |
| *End MP VII/Start LP* | | | | | -300 | -100 | -360 | 100 | 99.7 |
| OxA-30657 B 40 (2327,30) | -410 | -380 | -485 | -255 | -410 | -380 | -485 | -255 | 99.9 |
| OxA-30658 B 41 (2307,29) | -405 | -370 | -410 | -235 | -405 | -375 | -415 | -235 | 99.9 |
| OxA-30435 B 19 (2245,27) | -385 | -230 | -395 | -205 | -385 | -235 | -395 | -205 | 99.9 |
| OxA-30645 B 20 (2232,30) | -370 | -210 | -390 | -200 | -380 | -230 | -390 | -205 | 99.9 |
| OxA-30670 B 78 (2349,30) | -475 | -380 | -515 | -370 | -455 | -380 | -515 | -370 | 100 |
| OxA-30362 B 11 (2277,25) | -400 | -260 | -405 | -230 | -400 | -355 | -405 | -230 | 99.9 |
| OxA-30647 B 24 (2202,29) | -360 | -200 | -370 | -190 | -365 | -220 | -375 | -200 | 99.9 |
| OxA-30668 B 73 (2409,32) | -535 | -405 | -745 | -395 | -510 | -405 | -730 | -395 | 99.9 |
| OxA-30646 B 23 (2931,30) | -1210 | -1055 | -1220 | -1025 | -605 | -295 | -745 | -195 | 99.2 |
| **MP VII-VIII –Early Iron Age** | | | | | | | | | |
| *End EP VI/Start MP VII* | | | | | -760 | -505 | -765 | -455 | 98.8 |
| OxA-30648 B 25 (2498,29) | -770 | -550 | -790 | -535 | -790 | -620 | -795 | -555 | 99.6 |
| OxA-X-2583-34 B 21 (2511,25) | -775 | -555 | -790 | -540 | -790 | -600 | -795 | -550 | 99.7 |
| OxA-30649 B 27 (2499,30) | -770 | -550 | -790 | -535 | -790 | -620 | -795 | -555 | 99.7 |
| OxA-30650 B 29 (2553,29) | -800 | -595 | -805 | -550 | -800 | -760 | -805 | -565 | 99.9 |
| OxA-30651 B 30 (2558,29) | -800 | -595 | -805 | -550 | -800 | -760 | -810 | -585 | 99.9 |
| OxA-X-2590-19 B 56 (2513,30) | -775 | -555 | -795 | -540 | -795 | -625 | -795 | -550 | 99.7 |
| OxA-22380 (2516,26) | -780 | -555 | -795 | -540 |  | | | | |
| OxA-X-2559-13 B56 (2584,23) | -805 | -775 | -810 | -765 |  | | | | |
| B 56 pig bone (2554,18) | -795 | -770 | -805 | -590 | -795 | -770 | -805 | -590 | 100 |
| OxA-30653 B 33 (2632,29) | -820 | -795 | -840 | -780 | -820 | -795 | -840 | -780 | 100 |
| OxA-30656 B 36 (2469,29) | -755 | -535 | -770 | -430 | -775 | -650 | -785 | -540 | 99.6 |
| OxA-X-2583-28 B 12 (2628,24) | -815 | -795 | -830 | -785 | -815 | -795 | -830 | -785 | 100 |
| OxA-30659 (2632,30) | -820 | -790 | -845 | -775 |  | | | | |
| OxA-30660 (2637,29) | -820 | -795 | -890 | -785 |  | | | | |
| B 42 (2635,21) | -815 | -795 | -830 | -790 | -815 | -795 | -830 | -790 | 100 |
| OxA-30664 B 53 (2518,30) | -780 | -555 | -795 | -540 | -795 | -600 | -800 | -550 | 99.8 |
| OxA-30666 B 59 (2568,29) | -805 | -765 | -810 | -560 | -800 | -770 | -810 | -590 | 100 |
| OxA-X-2583-35 B 39* (2577,26) | -800 | -775 | -810 | -590 | -800 | -775 | -815 | -670 | 100 |
| OxA-22378 B 29 pig bone (2965,29) | -1225 | -1125 | -1270 | -1055 | -870 | -825 | -895 | -800 | 99.8 |
| **EP V – Later Bronze Age** | | | | | | | | | |
| *End EP IV/Start EP V* | | | | | -890 | -835 | -910 | -805 | 99.5 |
| OxA-30654 B 34 (2690,29) | -895 | -805 | -900 | -800 | -910 | -860 | -935 | -820 | 99.8 |
| OxA-30667 B 61 (2701,30) | -895 | -810 | -910 | -805 | -910 | -860 | -935 | -825 | 99.8 |
| OxA-30663 B 51 (2753,30) | -925 | -840 | -980 | -825 | -935 | -865 | -980 | -840 | 99.8 |
| OxA-30652 B 31 (2774,29) | -975 | -850 | -1000 | -840 | -975 | -895 | -995 | -855 | 99.8 |
| OxA-30655 B 35 (2767,30) | -975 | -845 | -1000 | -835 | -975 | -885 | -990 | -845 | 99.8 |
| OxA-30662 B 46 (2782,30) | -995 | -895 | -1005 | -840 | -975 | -900 | -1005 | -860 | 99.9 |
| OxA-22383 B 54 pig bone (2819,26) | -1005 | -930 | -1045 | -905 | -985 | -920 | -1020 | -900 | 99.9 |
| OxA-30665 B 55 (2815,31) | -1005 | -925 | -1055 | -895 | -985 | -915 | -1025 | -895 | 99.9 |
| OxA-30397 B 31* (2842,29) | -1045 | -935 | -1110 | -915 | -1005 | -925 | -1050 | -910 | 99.8 |
| OxA-25018 B 65 (2844,26) | -1045 | -940 | -1110 | -920 | -1000 | -925 | -1045 | -915 | 99.8 |
| OxA-30661 B 45 (2850,30) | -1055 | -940 | -1115 | -925 | -1005 | -925 | -1055 | -910 | 99.8 |
| OxA-22381 B 47 pig bone (2786,26) | -980 | -900 | -1010 | -845 | -975 | -900 | -1005 | -860 | 99.9 |
| OxA-30669 (2793,33) | -995 | -905 | -1020 | -840 |  | | | | |
| OxA-24047 (2868,26) | -1110 | -1000 | -1125 | -935 |  | | | | |
| B 76 (2840,21) | -1025 | -935 | -1060 | -915 | -1005 | -930 | -1045 | -915 | 99.8 |
| OxA-25016 (2789,26) | -980 | -905 | -1010 | -845 |  | | | | |
| OxA-25017 (2801,25) | -995 | -915 | -1020 | -890 |  | | | | |
| B 49 (2795,19) | -980 | -910 | -1005 | -900 | -975 | -910 | -1005 | -900 | 99.9 |
| OxA-25019 B 72 (2810,25) | -1000 | -925 | -1025 | -900 | -980 | -920 | -1010 | -900 | 99.9 |
| OxA-2436-53 B 47 (2936,25) | -1210 | -1110 | -1220 | -1045 | -1070 | -890 | -1100 | -855 | 98.6 |
| **EP III-IV Early Bronze Age** | | | | |  | | | | |
| *End EP II/Start EP III* | | | | | -1050 | -955 | -1130 | -940 | 98.9 |
| OxA-X-2438-16 B 45* (2958,29) | -1220 | -1125 | -1265 | -1055 | -1220 | -1125 | -1265 | -1060 | 99.2 |
| OxA-30363 B 41* (2976,25) | -1260 | -1130 | -1280 | -1115 | -1260 | -1125 | -1280 | -1115 | 99.4 |
| OxA-X-2438-17 B 47* (2978,31) | -1260 | -1125 | -1375 | -1085 | -1260 | -1125 | -1300 | -1085 | 99.2 |
| OxA-25014 B 43* (2984,26) | -1265 | -1130 | -1285 | -1115 | -1265 | -1130 | -1285 | -1115 | 99.3 |
| OxA-X-2593-43 B 74 (3061,30) | -1395 | -1275 | -1415 | -1230 | -1370 | -1260 | -1400 | -1220 | 99.2 |
| OxA-30671 B 33* (2792,30) | -980 | -905 | -1015 | -845 | -1235 | -960 | -1340 | -950 | 97.9 |
| OxA-X-2442-24 B 33* (3063,33) | -1395 | -1275 | -1415 | -1230 | -1365 | -1260 | -1405 | -1220 | 99.2 |
| **EP II Late Neolithic** | | | | |  | | | | |
| *End EP I/ Start EP II* |  |  |  |  | -1450 | -1310 | -1515 | -1270 | 98.9 |
| OxA-25015 B 44* (3242,26) | -1600 | -1455 | -1610 | -1440 | -1535 | -1450 | -1610 | -1435 | 100 |
| **EP I Early Neolithic** | | | | |  | | | | |
| *Start EP I* | | | | | -1675 | -1460 | -2080 | -1435 | 99.7 |

**S1 file Table F: Results of the Bayesian modelling from the site of Non Nok Tha.** See Caption for S1 file table E for details of the values in this table.

| **Non Nok Tha** | Unmodelled (BC/AD) | | | | Modelled (BC/AD) | | | | | Convergence values |
| --- | --- | --- | --- | --- | --- | --- | --- | --- | --- | --- |
|  | 68.2% probability | | 95.4% probability | | 68.2% probability | | | 95.4% probability | |  |
|  | *from* | *to* | *from* | *to* | *from* | | *to* | *from* | *to* |  |
| *End MP4-6* |  |  |  |  | -750 | | -665 | -775 | -525 | 98.5 |
| OxA-30391 B 24 (2414,27) | -520 | -405 | -735 | -400 | -750 | | -700 | -795 | -530 | 99.4 |
| OxA-30393 B 7 (2473,27) | -755 | -540 | -770 | -430 | -770 | | -710 | -790 | -625 | 99.6 |
| OxA-X-2586-16 B 80 (2505,31) | -770 | -550 | -790 | -535 | -785 | | -730 | -795 | -615 | 99.9 |
| OxA-X-2586-19 B 1 (2564,30) | -805 | -670 | -810 | -550 | -795 | | -765 | -810 | -665 | 100 |
| OxA-30394 B 17 (2566,27) | -800 | -765 | -810 | -565 | -795 | | -765 | -805 | -670 | 99.9 |
| OxA-30395 B 55 (2536,28) | -795 | -590 | -800 | -545 | -790 | | -750 | -805 | -595 | 99.9 |
| OxA-X-2586-15 B 85 (2570,27) | -800 | -770 | -810 | -585 | -795 | | -770 | -805 | -745 | 99.9 |
| **MP4-6** | | | | | |  | | | | |
| *Start MP4-6* | | | | | -815 | | -785 | -830 | -770 | 99.9 |
| OxA-X-2524-20 B 38 (2669,27) | -840 | -800 | -895 | -795 | -830 | | -805 | -855 | -795 | 100 |
| **MP3** | | | | | |  | | | | |
| *Start MP3* | | | | | -860 | | -810 | -890 | -800 | 100 |
| OxA-X-2524-21 B 62 (2717,25) | -895 | -830 | -910 | -810 | -895 | | -835 | -910 | -820 | 100 |
| **MP2** | | | | | |  | | | | |
| *Start MP2* | | | | | -940 | | -855 | -980 | -830 | 100 |
| OxA-30644 B 79 (2788,29) | -980 | -900 | -1010 | -845 | -980 | | -905 | -1015 | -870 | 100 |
| **EP3** | | | | | |  | | | | |
| *Start EP3* | | | | | -1060 | | -950 | -1110 | -905 | 100 |
| OxA-30361 B 78 (2879,26) | -1110 | -1010 | -1190 | -940 | -1115 | | -1030 | -1195 | -995 | 99.8 |
| OxA-30360 B 121 (2911,27) | -1190 | -1045 | -1210 | -1010 | -1190 | | -1050 | -1210 | -1020 | 99.9 |
| OxA-30396 B 35 (3028,28) | -1375 | -1225 | -1395 | -1195 | -1370 | | -1220 | -1395 | -1195 | 99.9 |
| OxA-30392 B 29 (3059,28) | -1390 | -1275 | -1410 | -1230 | -1375 | | -1265 | -1405 | -1230 | 99.8 |
| OxA-X-2586-18 B 94 (3102,30) | -1420 | -1300 | -1435 | -1285 |  | |  |  |  |  |
| OxA-X-2586-17 B 94 (3146,30) | -1490 | -1395 | -1500 | -1305 |  | |  |  |  |  |
| B 94 (3124,22) | -1435 | -1320 | -1445 | -1300 | -1425 | | -1305 | -1440 | -1290 | 99.9 |
| **EP1-2** | | | | | |  | | | | |
| *Start EP1-2* | | | | | -1425 | | -1305 | -1440 | -1290 | 99.9 |

**S1 file Table G: Results of the calibration and the Bayesian modelling from the site of Ban Lum Khao.** See Caption for S1 file table E for details of the values in this table.

| **Ban Lum Khao** | Unmodelled (BC/AD) | | | | Modelled (BC/AD) | | | | | | Convergence values |
| --- | --- | --- | --- | --- | --- | --- | --- | --- | --- | --- | --- |
|  | 68.2% probability | | 95.4% probability | | 68.2% probability | | | | 95.4% probability | |  |
|  | *from* | *to* | *from* | *to* | *from* | | *to* | | *from* | *to* |  |
| *End Bronze Age* | | | | | -795 | -755 | | | -800 | -710 | 99.8 |
| Wk-40460 B 4 (2564,20) | -795 | -770 | -805 | -595 | -800 | -780 | | | -805 | -765 | 99.9 |
| OxA-29143 B 84 (2608,26) | -810 | -790 | -820 | -770 | -810 | -790 | | | -820 | -775 | 100 |
| Wk-40463 B 27 (2624,20) | -810 | -795 | -820 | -790 | -810 | -795 | | | -825 | -785 | 99.9 |
| OxA-29139 B 30 (2640,27) | -825 | -795 | -845 | -785 | -825 | -795 | | | -845 | -785 | 99.9 |
| Wk-40459 B 3 (2720,22) | -895 | -830 | -910 | -815 | -895 | -830 | | | -910 | -815 | 99.9 |
| Wk-40470 B 89 (3115,20) | -1425 | -1320 | -1435 | -1300 | -1275 | -1125 | | | -1315 | -1120 | 99.7 |
| OxA-29141 B 89 (2753,27) | -920 | -840 | -975 | -825 | -920 | -840 | | | -980 | -825 | 99.9 |
| OxA-29144 B 107 (2729,26) | -900 | -835 | -925 | -815 | -900 | -835 | | | -925 | -815 | 99.9 |
| OxA-29174 B 61 (2748,27) | -915 | -840 | -975 | -825 | -915 | -840 | | | -975 | -820 | 99.9 |
| Wk-40461 B 7 (2762,20) | -930 | -845 | -975 | -835 | -930 | -845 | | | -975 | -835 | 99.9 |
| OxA-29140 B 59 (2775,28) | -975 | -850 | -1000 | -840 | -980 | -850 | | | -1005 | -840 | 99.9 |
| OxA-29142 B 52 (2766,29) | -975 | -845 | -995 | -835 | -970 | -845 | | | -995 | -835 | 99.9 |
| Wk-40467 B 52 (2783,20) | -975 | -900 | -1005 | -850 | -975 | -900 | | | -1005 | -850 | 99.9 |
| OxA-29175 B 62 (2802,26) | -995 | -915 | -1025 | -855 | -995 | -915 | | | -1025 | -855 | 99.9 |
| Wk-40462 B 10 (2824,20) | -1010 | -935 | -1030 | -915 | -1010 | -935 | | | -1035 | -910 | 99.9 |
| Wk-40465 B 29 (2896,20) | -1120 | -1040 | -1190 | -1005 | -1120 | -1040 | | | -1190 | -1005 | 99.9 |
| Wk-40471 B 90 (2918,20) | -1190 | -1050 | -1210 | -1030 | -1160 | -1050 | | | -1200 | -1025 | 99.9 |
| Wk-40464 B 28 (2925,20) | -1195 | -1055 | -1210 | -1045 | -1190 | -1055 | | | -1210 | -1045 | 99.9 |
| Wk-40466 B 40 (2950,20) | -1210 | -1125 | -1230 | -1055 | -1195 | -1120 | | | -1225 | -1055 | 99.9 |
| Wk-40468 B 70 (2932,20) | -1200 | -1090 | -1215 | -1050 |  | | | | | | |
| Wk-40469 B 70 (3014,20) | -1280 | -1220 | -1380 | -1130 |  | | | | | | |
| *Combine B 70* (2973,15) | -1225 | -1130 | -1260 | -1125 | -1220 | | -1125 | | -1265 | -1085 | 99.9 |
| **Bronze Age** | | | | |  | | | | | | |
| *Start Bronze Age* | | | | | -1290 | | -1175 | | -1330 | -1140 | 99.8 |
| Neolithic burials | | | | |  | | | | | | |
| *End Neolithic* | | | | | -1350 | | -1245 | | -1385 | -1200 | 99.8 |
| WK-4511 (3120,50) | -1445 | -1300 | -1500 | -1260 | -1380 | | -1280 | | -1420 | -1235 | 99.7 |
| Wk-4510 (3043,82) | -1415 | -1130 | -1495 | -1050 | -1380 | | -1275 | | -1410 | -1230 | 99.7 |
| Wk-4509 (3000,80) | -1385 | -1120 | -1430 | -1010 | -1380 | | -1275 | | -1410 | -1230 | 99.7 |
| Wk-4508 (3010,60) | -1385 | -1130 | -1415 | -1055 | -1380 | | -1275 | | -1405 | -1230 | 99.7 |
| Wk-4507 (3080,50) | -1410 | -1280 | -1450 | -1210 | -1380 | | -1280 | | -1410 | -1235 | 99.7 |
| **Neolithic** | | | | |  | | | | | | |
| *Start Neolithic* | | | | | -1415 | | | -1305 | -1470 | -1245 | 99.2 |

**S1 file Table H: Results of the Bayesian modelling of the Ban Na Di site, Area A (bottom) and Area B (top).** See caption to S1 file table E for details. Burial 15, excavated about 15m far from the rest of the burials, was not included because its stratigraphic position with MP2 or MP3 cannot be defined secured.

| **Ban Na Di** | Unmodelled (BC/AD) | | | | Modelled (BC/AD) | | | | Convergence values |
| --- | --- | --- | --- | --- | --- | --- | --- | --- | --- |
|  | 68.2% probability | | 95.4% probability | | 68.2% probability | | 95.4% probability | |  |
|  | *from* | *to* | *from* | *to* | *from* | *to* | *from* | *to* |  |
| *End MP1b - Area B* | | | | | -790 | -500 | -795 | 120 | 98.2 |
| OxA-X-2524-27 B 39 (2534,25) | -795 | -590 | -800 | -545 | -795 | -590 | -800 | -550 | 99.7 |
| OxA-28430 B 42 (2552,31) | -800 | -590 | -805 | -550 | -800 | -590 | -805 | -550 | 99.8 |
| **MP1b - Area B** | | | | |  | | | | |
| *Start MP1b - Area B* | | | | | -935 | -600 | -1420 | -575 | 99.1 |
| **Ban Na Di - Area B** | | | | |  | | | | |
| *End MP1c - Area A* | | | | | -415 | -165 | -495 | 420 | 99.5 |
| OxA-28126 B 9 (2339,25) | -410 | -390 | -480 | -365 | -410 | -385 | -490 | -370 | 99.9 |
| **MP1c** | | | | |  | | | | |
| *Start MP1c - Area A* |  | | | | -610 | -400 | -740 | -390 | 99.8 |
| OxA-28106 B 10 (2497,27) | -765 | -550 | -780 | -535 | -690 | -550 | -770 | -540 | 99.9 |
| OxA-30416 B 11 (2455,27) | -750 | -485 | -755 | -410 | -745 | -530 | -760 | -485 | 99.8 |
| OxA-30415 B 12 (2515,27) | -775 | -555 | -795 | -540 | -690 | -555 | -780 | -540 | 99.9 |
| **MP1b** | | | | | | | | | |
| *Start MP1b - Area A* | | | | | -795 | -680 | -800 | -590 | 99.9 |
| OxA-30377 B 28 (2594,24) | -805 | -785 | -810 | -770 |  | | | | |
| OxA-30414 B 28 (2559,29) | -805 | -595 | -805 | -550 |  | | | | |
| B 28 (2580,19) | -800 | -780 | -805 | -770 | -800 | -780 | -810 | -765 | 100 |
| **MP1a** | | | | |  | | | | |
| *Start MP1a - Area A* | | | | | -930 | -775 | -1300 | -765 | 99.7 |
| **Ban Na Di - Area A** | | | | | | | | | |

**S1 file Table I: Results of the Bayesian outlier analysis for Ban Chiang.** Prior probabilities are the outlier probabilities set before the model run, whilst the posterior probabilities denote out outlying each determination in within the overall sequence. A posterior outlier probability of 50% means that that determination is left out of the model in half of the total run. The outlier models used are shown in the table as well. The prior outlier probability for most determinations in the model was set at 0.05. The table lists the prior and posterior outlier results and well as the type of model used [38], it can be seen that there are only two outliers of significance (OxA-30646 and OxA-22378) which are 100% outliers and therefore not included in the modelling runs. There is a further date (OxA-X-2436–53) which is 62% likely to be an outlier and a final determination (OxA-30671) that is 40% likely outlying. The combined data (duplicate dates of the same burial) are shown in italic. In asterisk are the dates from the 1974 excavation, all others are from the 1975 season.

| **Element** | **Prior** | **Posterior** | **Model** | **Type** | **Element** | **Prior** | **Posterior** | **Model** | **Type** |
| --- | --- | --- | --- | --- | --- | --- | --- | --- | --- |
| OxA-25015 B 44* | 5 | 2 | General | t | OxA-X-2583-35 B 39* | 5 | 1 | General | t |
| OxA-X-2442-24 B 33* | 5 | 2 | General | t | OxA-30666 B 59 | 5 | 1 | General | t |
| OxA-30671 B 33* | 5 | 40 | General | t | OxA-30664 B 53 | 5 | 2 | General | t |
| OxA-X-2593-43 B 74 | 5 | 2 | General | t | B 42 | 5 | 1 | General | t |
| OxA-25014 B 43* | 5 | 2 | General | t | *OxA-30660* | 5 | 3 | SSimple | s |
| OxA-X-2438-17 B 47* | 5 | 2 | General | t | *OxA-30659* | 5 | 3 | SSimple | s |
| OxA-30363 B 41* | 5 | 2 | General | t | OxA-X-2583-28 B 12* | 5 | 1 | General | t |
| OxA-X-2438-16 B 45* | 5 | 2 | General | t | OxA-30656 B 36 | 5 | 3 | General | t |
| OxA-2436-53 B 47 | 5 | 62 | General | t | OxA-30653 B 33 | 5 | 1 | General | t |
| OxA-25019 B 72 | 5 | 1 | General | t | B 56 pig bone from burial | 5 | 1 | General | t |
| B 49 | 5 | 1 | General | t | *OxA-X-2559-13 B 56* | 5 | 13 | SSimple | s |
| *OxA-25017* | 5 | 3 | SSimple | s | *OxA-22380* | 5 | 8 | SSimple | s |
| *OxA-25016* | 5 | 3 | SSimple | s | OxA-X-2590-19 B 56 | 5 | 2 | General | t |
| B 76 | 5 | 1 | General | t | OxA-30651 B 30 | 5 | 1 | General | t |
| *OxA-24047* | 5 | 8 | SSimple | s | OxA-30650 B 29 | 5 | 1 | General | t |
| *OxA-30669* | 5 | 6 | SSimple | s | OxA-30649 B 27 | 5 | 2 | General | t |
| OxA-22381 B 47 pig bone | 5 | 1 | General | t | OxA-X-2583-34 B 21* | 5 | 2 | General | t |
| OxA-30661 B 45 | 5 | 2 | General | t | OxA-30648 B 25 | 5 | 2 | General | t |
| OxA-25018 B 65 | 5 | 1 | General | t | OxA-30646 B 23 | 5 | 100 | General | t |
| OxA-30397 B 31* | 5 | 1 | General | t | OxA-30668 B 73 | 5 | 2 | General | t |
| OxA-30665 B 55 | 5 | 1 | General | t | OxA-30647 B 24 | 5 | 2 | General | t |
| OxA-22383 B 54 pig bone | 5 | 1 | General | t | OxA-30362 B 11* | 5 | 2 | General | t |
| OxA-30662 B 46 | 5 | 1 | General | t | OxA-30670 B 78 | 5 | 2 | General | t |
| OxA-30655 B 35 | 5 | 1 | General | t | OxA-30645 B 20 | 5 | 2 | General | t |
| OxA-30652 B 31 | 5 | 1 | General | t | OxA-30435 B 19 | 5 | 2 | General | t |
| OxA-30663 B 51 | 5 | 1 | General | t | OxA-30658 B 41 | 5 | 2 | General | t |
| OxA-30667 B 61 | 5 | 3 | General | t | OxA-30657 B 40 | 5 | 2 | General | t |
| OxA-30654 B 34 | 5 | 5 | General | t | OxA-30433 Burial 6 | 5 | 2 | General | t |
| OxA-22378 B 29 pig bone | 5 | 100 | SSimple | s | OxA-30434 Burial 9 | 5 | 2 | General | t |

**S1 file Table J: Outlier detection results from the site of Non Nok Tha.** See caption for S1 file table I for details.

| **Element** | **Prior** | **Posterior** | **Model** | **Type** |
| --- | --- | --- | --- | --- |
| Burial 94 | 5 | 5 | General | t |
| OxA-X-2586-17 B 94 | 5 | 7 | SSimple | s |
| OxA-X-2586-18 B 94 | 5 | 3 | SSimple | s |
| OxA-30392 B 29 | 5 | 4 | General | t |
| OxA-30396 B 35 | 5 | 4 | General | t |
| OxA-30360 B 121 | 5 | 4 | General | t |
| OxA-30361 B 78 | 5 | 4 | General | t |
| OxA-30644 B 79 | 5 | 3 | General | t |
| OxA-X-2524-21 B 62 | 5 | 3 | General | t |
| OxA-X-2524-20 B 38 | 5 | 3 | General | t |
| OxA-X-2586-15 B 85 | 5 | 3 | General | t |
| OxA-30395 B 55 | 5 | 3 | General | t |
| OxA-30394 B 17 | 5 | 3 | General | t |
| OxA-X-2586-19 B1 | 5 | 3 | General | t |
| OxA-X-2586-16 B80 | 5 | 4 | General | t |
| OxA-30393 B7 | 5 | 4 | General | t |
| OxA-30391 B24 | 5 | 9 | General | t |

**S1 file Table K: Outlier detection results from the site of Ban Lum Khao.** See caption for S1 file table J for details.

| Wk-4507 | 100 | 100 | Charcoal | t |
| --- | --- | --- | --- | --- |
| Wk-4508 | 100 | 100 | Charcoal | t |
| Wk-4509 | 100 | 100 | Charcoal | t |
| Wk-4510 | 100 | 100 | Charcoal | t |
| WK-4511 | 100 | 100 | Charcoal | t |
| B 70 | 5 | 4 | General | t |
| Wk-40469 B70 | 5 | 39 | SSimple | s |
| Wk-40468 B70 | 5 | 15 | SSimple | s |
| Wk-40466 B40 | 5 | 4 | General | t |
| Wk-40464 B28 | 5 | 4 | General | t |
| Wk-40471 B90 | 5 | 4 | General | t |
| Wk-40465 B29 | 5 | 4 | General | t |
| Wk-40462 B10 | 5 | 4 | General | t |
| OxA-29175 B62 | 5 | 4 | General | t |
| Wk-40467 B52 | 5 | 4 | General | t |
| OxA-29142 B52 | 5 | 4 | General | t |
| OxA-29140 B59 | 5 | 4 | General | t |
| Wk-40461 B7 | 5 | 4 | General | t |
| OxA-29174 B61 | 5 | 4 | General | t |
| OxA-29144 B107 | 5 | 4 | General | t |
| OxA-29141 B89 | 5 | 5 | SSimple | s |
| Wk-40470 B89 | 50 | 100 | SSimple | s |
| Wk-40459 B3 | 5 | 4 | General | t |
| OxA-29139 B30 | 5 | 3 | General | t |
| Wk-40463 B27 | 5 | 4 | General | t |
| OxA-29143 B84 | 5 | 3 | General | t |
| Wk-40460 B4 | 5 | 4 | General | t |

**S1 file Table L: Outliers from the Ban Na Di model.** See caption to S1 file Table I for details.

| **Element** | **Prior** | **Posterior** | **Model** | **Type** |
| --- | --- | --- | --- | --- |
| Burial 28 | 5 | 3 | General | t |
| OxA-30414 B 28 | 5 | 3 | SSimple | s |
| OxA-30377 B 28 | 5 | 5 | SSimple | s |
| OxA-30415 B 12 | 5 | 3 | General | t |
| OxA-30416 B 11 | 5 | 3 | General | t |
| OxA-28106 B 10 | 5 | 3 | General | t |
| OxA-28126 B 9 | 5 | 4 | General | t |
| OxA-28430 B 42 | 5 | 3 | General | t |
| OxA-X-2524-27 B 39 | 5 | 3 | General | t |

**S1 file Table M: %N measurements of bone from the Non Nok Tha site.** Anything below 0.8-1% is very unlikely to contain intact collagen enough for a radiocarbon determination. Human bones from burial contexts indicated with an asterisk (*) underwent collagen extraction but either no collagen was found or not enough for a radiocarbon determination.

| **Burial no.** | **wt %N** |  | **Burial no.** | **wt %N** |
| --- | --- | --- | --- | --- |
| 1 | 0.74 |  | 57 * | 1.02 |
| 2 | 0.07 |  | 58 | 0.16 |
| 3 | 0.47 |  | 59 | 0.06 |
| 4 | 0.45 |  | 60 | 0.48 |
| 5 | 0.58 |  | 61 | 0.46 |
| 7 | 0.79 |  | 62 | 0.14 |
| 9 | 0.38 |  | 64 * | 0.64 |
| 10 * | 0.70 |  | 67 | 0.33 |
| 11 | 0.59 |  | 68 | 0.28 |
| 12 * | 0.61 |  | 70 | 0.43 |
| 14 | 0.24 |  | 71 | 0.15 |
| 15 * | 0.44 |  | 73 * | 0.67 |
| 16 | 0.04 |  | 74 | 0.28 |
| 17 | 1.23 |  | 75 | 0.20 |
| 18 | 0.04 |  | 76 | 0.48 |
| 20 | 0.45 |  | 77 | 0.03 |
| 21 | 0.55 |  | 78 | 1.67 |
| 22 | 0.46 |  | 79 | 0.72 |
| 23 | 0.28 |  | 80 | 0.22 |
| 24 | 1.06 |  | 80 | 0.85 |
| 25 | 0.53 |  | 81 * | 0.75 |
| 26 | 0.54 |  | 82 | 0.13 |
| 27 | 0.20 |  | 83 | 0.35 |
| 28 * | 0.10 |  | 85 | 1.01 |
| 29 | 1.46 |  | 86 | 0.50 |
| 31 | 0.34 |  | 87 | 0.12 |
| 32 * | 0.68 |  | 88 | 0.23 |
| 33 | 0.28 |  | 89 | 0.42 |
| 34 | 0.16 |  | 90 | 0.27 |
| 35 | 1.98 |  | 91 | 0.07 |
| 36 | 0.43 |  | 92 | 0.40 |
| 37 | 0.57 |  | 94 | 0.72 |
| 38 | 0.30 |  | 95 | 0.12 |
| 39 * | 0.74 |  | 96 | 0.03 |
| 40 * | 0.61 |  | 103 | 0.35 |
| 41 | 0.35 |  | 108 * | 0.84 |
| 42 | 0.39 |  | 112 | 0.46 |
| 43 | 0.13 |  | 116 * | 0.76 |
| 45 * | 0.97 |  | 117 | 0.31 |
| 46 | 0.12 |  | 121 | 0.83 |
| 47 | 0.32 |  | 124 | 0.29 |
| 48 | 0.16 |  | 125 | 0.11 |
| 49 | 0.47 |  | M101 | 0.63 |
| 51 | 0.21 |  | M107 | 0.07 |
| 52 | 0.65 |  | M110A | 0.54 |
| 55 | 0.88 |  | M111 | 0.68 |
| 56 | 0.27 |  | M114 | 0.44 |
